# A Piezo‐Mimetic Ionic Hydrogel Harnessing Joint Motion for Cartilage Repair

**DOI:** 10.1002/advs.75611

**Published:** 2026-05-10

**Authors:** Chenyuan Gao, Xinyu Wang, Zhifeng Wu, Xinya Zhang, Xiao Geng, Ti Zhang, Yue Wang, Hongwei Xia, Jun Zhang, Xianbo Qiu, Cuiru Sun, Yingjie Yu, Wenli Dai, Hua Tian, Qing Cai

**Affiliations:** ^1^ Engineering Research Center of Bone and Joint Precision Medicine Ministry of Education Department of Orthopaedics Peking University Third Hospital Beijing China; ^2^ State Key Laboratory of Organic‐Inorganic Composites Beijing Laboratory of Biomedical Materials Beijing University of Chemical Technology Beijing China; ^3^ Institute of Microfluidic Chip Development in Biomedical Engineering School of Information Science and Technology Beijing University of Chemical Technology Beijing China; ^4^ Department of Mechanics Tianjin University Tianjin China; ^5^ Institute of Sports Medicine Beijing Key Laboratory of Sports Injuries Peking University Third Hospital Beijing China

## Abstract

Articular cartilage regeneration remains challenging due to its avascular architecture and limited intrinsic repair capacity. Here, we present a piezo‐mimetic ionic hydrogel (PSG‐Mg_c_) that harnesses endogenous joint motion to orchestrate cartilage repair through mechano‐electrical coupling. The hydrogel integrates a freeze‐thaw crosslinked poly(vinyl alcohol) (PVA) backbone for mechanical resilience with sodium alginate (SA) and carboxyl‐enriched acylated gelatin (Acy‐Gel) to construct a hydrated, ion‐permissive network capable of dynamic Mg^2+^ coordination. Unlike conventional electron‐based piezoelectric materials, PSG‐Mg_c_ transduces mechanical deformation into localized ionic currents via deformation‐induced asymmetric ion migration, while simultaneously enabling sustained Mg^2+^ delivery to modulate cellular metabolism. This dual mechano‐electrical coupling and biochemical regulation enhance chondrocyte activity and promote extracellular matrix synthesis in vitro. In anatomically distinct trochlear and femoral condylar defect models, the hydrogel exhibits load‐adaptive functionality, generating amplified bioelectric cues under higher mechanical stress and achieving near‐native cartilage restoration. By coupling mechanical energy harvesting with ion‐mediated electrochemical signaling, PSG‐Mg_c_ establishes a new paradigm that shifts cartilage repair from passive scaffolding toward active mechano‐electrical coupling regeneration.

## Graphical abstract

A piezo‐mimetic ionic hydrogel converts mechanical deformation into bioactive ionic currents via stress‐induced asymmetric ion migration. Coupled with sustained Mg^2+^ release, this mechano‐electrochemical platform bridges mechanical loading and cellular signaling, enabling load‐adaptive stimulation and enhanced cartilage regeneration.

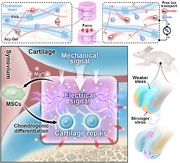

## 1 Introduction

Articular cartilage repair remains a major clinical challenge, largely because current regenerative strategies fail to recreate the dynamic biophysical cues that govern chondrocyte function in vivo [1, 2, 3]. Despite decades of development, current clinical interventions, including microfracture, osteochondral grafting, and autologous chondrocyte implantation, cannot consistently regenerate durable hyaline cartilage and frequently result in fibrocartilage formation or long‐term failure [4, 5, 6, 7]. A key limitation of these approaches is their insufficient consideration of the unique mechanical microenvironment of articular cartilage, which plays a critical role in regulating cellular behavior and tissue homeostasis [8]. In native cartilage, chondrocytes reside in a mechanically dynamic environment, where compressive and cyclic loading is tightly coupled with electrochemical signaling processes that regulate ion transport, cellular metabolism, and extracellular matrix synthesis [9, 10, 11]. However, most existing cartilage scaffolds primarily serve as passive structural supports and lack the ability to actively harness or modulate these mechanical cues, thereby limiting their regenerative potential [12, 13, 14].

Piezoelectric materials have recently emerged as promising candidates for converting mechanical deformation into electrical stimulation to regulate chondrocyte behavior [15, 16]. Although inorganic ceramics and polarized polymers can generate electrical potentials under stress [17], their rigid nature, limited ionic permeability, and reliance on electron‐based conduction fundamentally differ from the ion‐mediated electrochemical communication that dominates cartilage physiology [18, 19]. Importantly, chondrocyte‐chondrocyte communication and mechanotransduction are intrinsically governed by dynamic ionic exchanges rather than static electronic currents [20]. Mechanical loading in cartilage induces spatiotemporal redistribution of ions, alters membrane potentials, and activates mechanosensitive ion channels, thereby orchestrating downstream signaling pathways essential for tissue homeostasis and regeneration [21]. The inability of conventional piezoelectric materials to participate in or amplify such ion‐driven processes severely limits their capacity to establish sustained, physiologically relevant interactions with the native cartilage microenvironment [22]. Consequently, these materials often exhibit suboptimal biointegration, transient stimulation effects, and limited long‐term efficacy in cartilage repair applications [23]. Collectively, these limitations highlight a critical need for the development of next‐generation mechano‐electrical materials that transcend simple force‐to‐voltage conversion and instead emulate the ion‐mediated communication paradigms intrinsic to cartilage tissue [24]. Materials capable of coupling mechanical deformation with ion transport, electrochemical signaling, and dynamic cellular interactions are urgently required to achieve truly biomimetic regulation of chondrocyte behavior and effective cartilage regeneration.

The electromechanical behavior of native cartilage has long been recognized not as a classical piezoelectric process, but as an electrokinetic phenomenon intrinsic to hydrated, charge‐rich soft tissues [25]. As a load‐bearing and lubricating tissue, cartilage converts mechanical deformation into electrical signals through stress‐induced redistribution of mobile ions within its structurally graded extracellular matrix. This mechano‐electrical coupling originates from the movement of counterions in a highly hydrated polyelectrolyte network, generating transient electrical potentials that regulate fluid transport, nutrient diffusion, and local cellular signaling [26]. Importantly, such mechano‐electrical coupling does not rely on electron polarization, but instead arises from mechanically gated ionic migration and electrochemical gradient formation. This distinction reveals a fundamental mismatch between native cartilage electromechanics and conventional piezoelectric materials. While traditional piezoelectric ceramics and polymers generate electrical outputs through polarization of bound charges [27, 28], cartilage produces bioelectrical signals via ion transport in response to deformation [29]. These two mechanisms are mechanistically orthogonal, despite producing superficially similar electrical readouts. Consequently, replicating cartilage electromechanical function requires materials that can support ion mobility, electrochemical coupling, and deformation‐driven ionic flux, rather than simply converting stress into voltage. Despite its central role in cartilage physiology, this piezo‐mimetic, ion‐driven electromechanical mechanism has been largely overlooked in the design of cartilage repair materials. Most existing approaches remain confined to electron based piezoelectric paradigms, leaving a critical gap between material generated electrical cues and biologically relevant mechano‐electrical coupling. This gap highlights the necessity of developing mechanically responsive, ion conductive hydrogel systems capable of emulating the electrokinetic behavior of chondrocytes, thereby providing a rational foundation for the design of piezo‐mimetic ionic biomaterials for cartilage regeneration.

Ionic conductive hydrogels provide a compelling and biomimetic platform to recreate this piezo‐mimetic behavior [30]. Upon mechanical deformation, charged polymer networks undergo asymmetric migration of cations and anions, generating localized ionic currents and transient electrical fields that closely resemble the endogenous electrokinetic signals produced in native cartilage during joint motion [31]. These charged groups primarily stem from covalently attached anionic groups (e.g., ─COO^−^, ─SO_3_^−^) or cationic groups on polymer chains, their corresponding mobile counterions (e.g., Na^+^, Cl^−^), as well as extra ionic species derived from dissolved salts (e.g., CaCl_2_, MgCl_2_) [32, 33]. Unlike conventional piezoelectric materials, which rely on rigid crystalline structures and often suffer from limited biocompatibility and inefficient signal transmission [34, 35], ionic conductive hydrogels leverage hydrated polymer networks to enable soft, reversible, and dynamically tunable signal generation [36]. Importantly, such ionic conduction is inherently biocompatible, dynamically responsive, and capable of coupling mechanical loading with biochemical signaling, features unattainable with conventional piezoelectric scaffolds.

**SCHEME 1:**
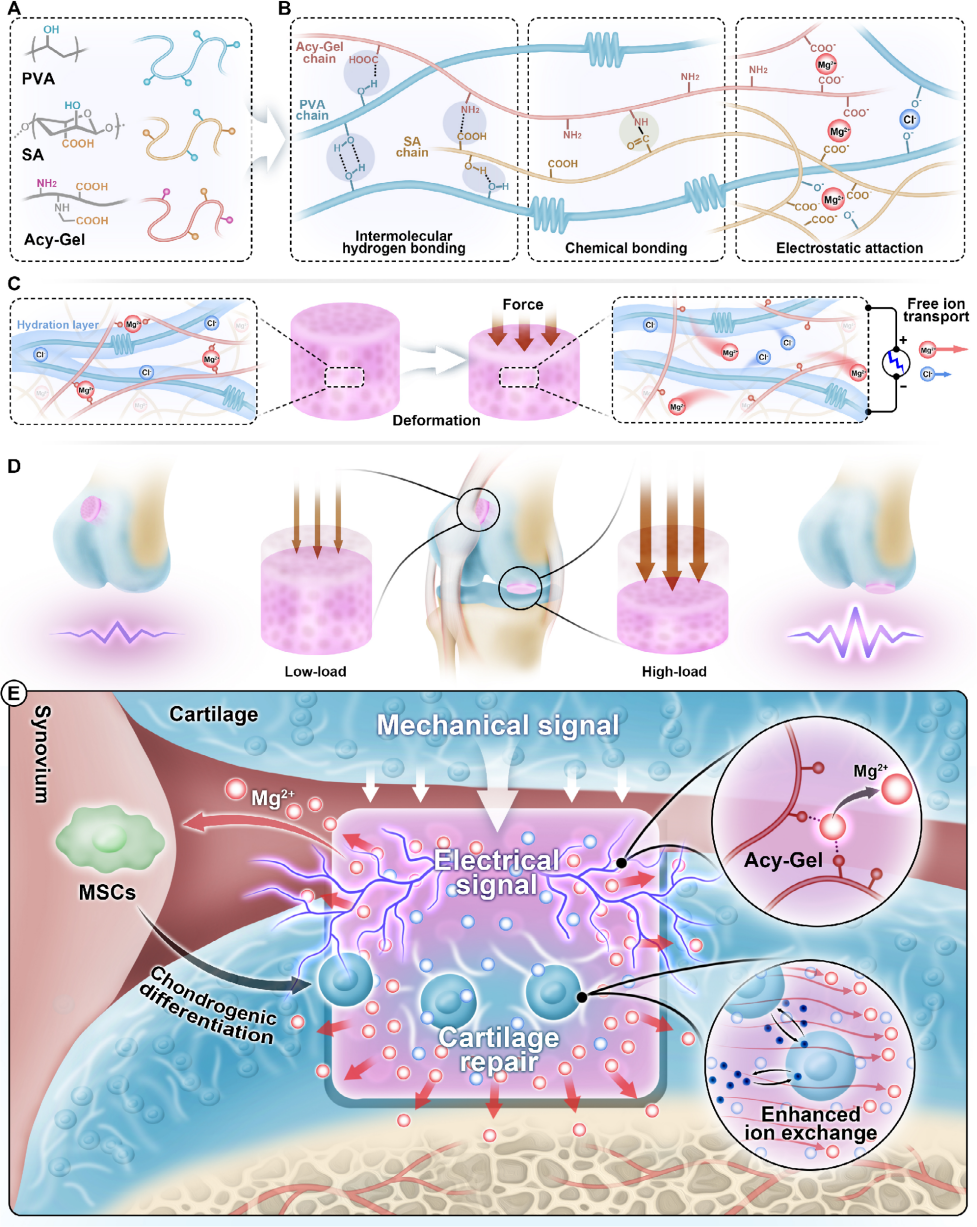
Design and piezo‐mimetic mechanism of the PVA/SA/Acy‐Gel (PSG) hydrogel for cartilage regeneration. (A) Chemical structures and functional components of the hydrogel system, including poly(vinyl alcohol) (PVA), sodium alginate (SA), and multi‐carboxyl‐modified acylated gelatin (Acy‐Gel). (B) Schematic illustration showing the molecular‐level interactions within the PSG hydrogel. Intermolecular hydrogen bonding between freeze‐thaw induced folded PVA chains and SA establishes a physically crosslinked and hydrated backbone. EDC/NHS‐mediated chemical bonding between amino and carboxyl groups on Acy‐Gel forms covalent crosslinks that reinforce the polymer network. The carboxyl‐rich SA and Acy‐Gel chains further provide electrostatic coordination sites for divalent Mg^2+^ ions, while Cl^−^ ions are confined within the hydrogen‐bonded PVA microdomains. (C) Under mechanical loading, deformation of the hydrogel induces asymmetric transport of cations and anions due to their distinct mobilities within the hydrated polymer network, generating a piezo‐mimetic ionic current. (D) The magnitude of piezo‐ionic signal generation is load‐dependent, enabling adaptive mechano‐electrical coupling under low‐ and high‐stress joint environments. (E) In a rabbit cartilage defect model, mechanical stimulation generated by joint motion is transduced into localized ionic and electrical signals within the hydrogel, enhancing Mg^2+^‐mediated ion exchange, mimicking physiological ion‐based intercellular communication, and thereby promoting stem cell chondrogenic differentiation, facilitating cell‐cell ionic crosstalk, and driving in situ cartilage regeneration.

Herein, we propose a piezo‐mimetic ionic hydrogel that harnesses joint motion as an endogenous energy source to drive cartilage regeneration through synergistic mechano‐electrical coupling. As illustrated in Scheme 1A,B, this system is constructed by integrating freeze‐thaw crosslinked poly(vinyl alcohol) (PVA) as a mechanically robust and hydrogen‐bonded backbone, sodium alginate (SA) as an anionic polyelectrolyte network capable of multivalent cation coordination, and acylated gelatin (Acy‐Gel) as a carboxyl‐enriched macromolecular matrix that provides both chemical crosslinking sites and dynamic ion‐chelation capacity. Intermolecular hydrogen bonding between PVA and SA establishes a hydrated, deformable network, while EDC/NHS‐mediated covalent bonding within Acy‐Gel further reinforces scaffold integrity, yielding a mechanically resilient yet ion‐permeable composite hydrogel (Scheme 1B). As shown in Scheme 1C, the highly hydrated microenvironment of the composite enables the coordinated incorporation of bioactive Mg^2+^ ions, which are electrostatically confined by SA and Acy‐Gel while remaining dynamically exchangeable within the polymer network. Upon mechanical loading, deformation of the hydrogel disrupts local ion confinement and hydration layers, inducing asymmetric migration of mobile ions and generating localized ionic currents through deformation‐gated ion transport rather than electron polarization. This mechano‐electrical coupling process underlies the piezo‐mimetic behavior of the PSG‐Mg_c_ hydrogel, allowing mechanical stress to be directly transduced into biologically relevant electrochemical signals. In vivo, we employed a rabbit cartilage defect model, which offers the well‐defined load‐bearing heterogeneity, to rigorously evaluate mechano‐electrical coupling cartilage regeneration. By creating anatomically distinct defects in the trochlear groove and femoral condyle, we established low‐ and high‐mechanical‐loading environments within the same joint, enabling direct assessment of load‐dependent regenerative outcomes (Scheme 1D). Under physiological joint motion, the piezo‐mimetic ionic hydrogel not only promoted robust cartilage regeneration but also achieved near‐native structural restoration in the high‐load femoral condylar region (Scheme 1E). This work establishes a fundamentally new paradigm for cartilage repair, one that shifts from passive mechanical support to active mechano‐electrical coupling, providing a blueprint for intelligent biomaterials that harness endogenous joint motion to drive tissue regeneration.

## 2 Results and Discussion

### 2.1 Design and Characterization of Piezo‐Mimetic Ionic Hydrogels

In vivo cellular signal transduction primarily relies on the transmission of ionic signals [37, 38]. To mimic ion‐electron signal conduction under physiological conditions, we designed a piezo‐mimetic ionic hydrogel system with intrinsic piezoelectric properties. Alginate is well known for its ability to coordinate divalent cations (Ca^2+^, Zn^2+^, etc.) through ionic crosslinking [39, 40]. However, the dense distribution of carboxyl groups along its linear alginate backbone leads to an ion‐shielding effect, reducing the number of accessible coordination sites and increasing electrostatic repulsion [41], which stiffens the polymer chains and compromises mechanical toughness, hence rendering SA‐based hydrogels unsuitable for load‐bearing cartilage repair [42].

**FIGURE 1:**
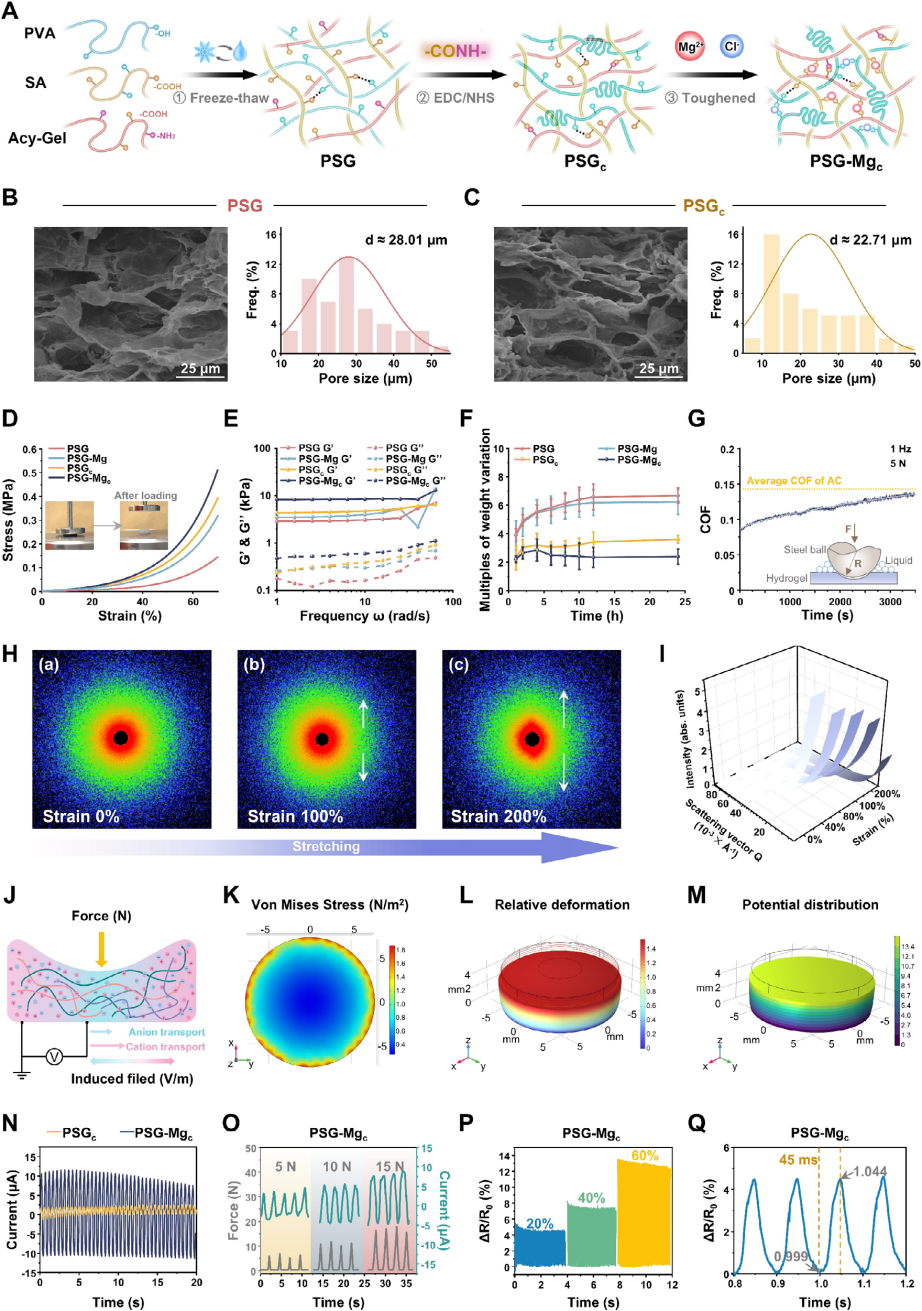
Preparation and characterization of the piezo‐mimetic ionic hydrogels. (A) Schematic illustration of the ion‐induced cross‐linking and chemical crosslinked networks within the hydrogels to form PSG, PSG_c_, PSG‐Mg, and PSG‐Mg_c_. (B,C) Cross‐sectional SEM images and corresponding pore‐size distributions of PSG and PSG_c_ hydrogels. (D) Compressive stress–strain curves of the hydrogels. (E) Rheological analysis of the hydrogels. G’ represents the storage modulus, and G″ represents the loss modulus. (F) Swelling behavior of the hydrogels. (G) Friction coefficients of PSG‐Mg_c_ hydrogel measured at 1 Hz and 5 N in physiological saline as the lubricants. (H) In situ SAXS patterns of PSG‐Mg_c_ hydrogel under tensile strains of 0%, 100%, and 200%. (I) Integrated spectra of SAXS profiles recorded at 0%, 40%, 80%, 100%, and 200% strain. (J) Schematic illustration of the mechano‐electrical conversion in the piezo‐mimetic ionic hydrogel. (K) COSMOL finite element analysis showing the stress distribution of the PSG‐Mg_c_ hydrogel under compression. (L) Stress distribution at different layers during deformation. (M) Electrical potential distribution generated upon deformation. (N) Output current responses of PSG_c_ and PSG‐Mg_c_ hydrogels under cyclic compression at 20% strain. (O) Synchronized current and stress signals of PSG‐Mg_c_ under compression of 5, 10, and 15 N. (P) Cyclic strain‐sensing performance of PSG‐Mg_c_ at 20%, 40%, and 60% strain. (Q) Dynamic response behavior of PSG‐Mg_c_ under 20% compressive deformation. Results are presented as mean ± SD (*n* ≥ 3).

To overcome these limitations, we introduced acylated gelatin (Acy‐Gel) as a secondary carboxyl‐rich polymer. Compared with SA, Acy‐Gel offers: (i) a lower local charge density, alleviating ion‐shielding; (ii) flexible peptide backbones that improve network compliance; and (iii) inherent cell‐adhesive motifs that promote cytocompatibility [43]. Both SA and Acy‐Gel form extensive hydrogen bonding with PVA, enabling the construction of a robust interpenetrating network that theoretically supports continuous ion migration. Acy‐Gel was first synthesized (Figure S1) and subsequently integrated with PVA and SA to construct an interpenetrating network, forming the PSG hydrogel (Figure 1A). PVA/Acy‐Gel (PG) and PVA/SA (PS) hydrogels were prepared as controls. XPS analysis confirmed that PSG exhibited the highest intensities of carbonyl (C = O, ∼287.9 eV) and carboxylate (O─C = O, ∼288.7 eV) peaks, reflecting the synergistic contribution of SA and Acy‐Gel and yielding the highest surface density of carboxyl functionalities (Figure S2).

To activate ionic conduction, PSG was first ionically crosslinked with Ca^2+^. Considering our previous finding that Mg^2+^ exerts superior pro‐chondrogenic regulation, Mg^2+^ was subsequently incorporated to yield PSG‐Mg [44, 45, 46]. Hydrogels equilibrated in PBS without divalent ions served as controls. The resulted hydrogels, crosslinked via different ionic interactions, were then systematically compared in terms of toughness, mechanical strength, resilience, and ion‐binding capacity (Figures S3–S6, and Movie S1). The results revealed that SA preferentially coordinates Ca^2+^, which possesses a larger ionic radius, whereas Acy‐Gel exhibits a stronger affinity toward the smaller Mg^2+^, indicating complementary ion‐binding behaviors within the PSG network. Further analyses were performed to assess ionic conductivity (Figure S7), output current response (Figure S8), and the biocompatibility of PS, PG, and PSG hydrogels equilibrated in different ionic solutions (Figure S9). Detailed analyses are provided in Texts S1–S5. Collectively, these results identified the PSG‐Mg hydrogel as the optimized formulation, demonstrating favorable potential for cartilage repair applications.

Although Mg^2+^ confers favorable biological activity, Mg‐carboxyl coordination alone provides relatively weak physical crosslinking and is insufficient to ensure long‐term structural stability. To reinforce the network, PSG‐Mg was therefore subjected to EDC/NHS coupling to introduce covalent amide bonds, yielding the dual‐crosslinked hydrogel PSG‐Mg_c_. In parallel, PSG treated with EDC/NHS (denoted PSG_c_) was prepared as a control. SEM imaging confirmed that this secondary covalent crosslinking did not disrupt the interconnected porous architecture of the hydrogels (Figure 1B,C). Mechanical characterization further revealed a pronounced strengthening effect: both compression and rheological tests showed that PSG_c_ and PSG‐Mg_c_ exhibited substantially higher compressive stress and storage modulus than their non‐covalently crosslinked counterparts (PSG and PSG‐Mg), with PSG‐Mg_c_ achieving the highest mechanical performance while still maintaining excellent deformability (compressive strain > 60%) (Figure 1D,E).

Consistently, swelling experiments over 24 h demonstrated that PSG_c_ and PSG‐Mg_c_ retained superior structural integrity, showing only ∼2‐fold mass increase compared with ∼7‐fold swelling for PSG and PSG‐Mg (Figure 1F). These hydrogels also displayed enhanced resistance to degradation, with PSG‐Mg_c_ exhibiting the lowest degradation rate (Figure S10). This improvement arises from the synergistic stabilization provided by covalent amide crosslinks together with physical ionic and hydrogen‐bonding interactions. The resulting enhancement in shape stability and prolonged structural persistence is expected to be highly advantageous for subsequent in vitro and in vivo cartilage repair applications.

Given the ultralow friction nature of articular cartilage [47], the tribological performance of PSG‐Mg_c_ was systematically evaluated using a ball‐on‐disk tribometer in a reciprocating sliding mode (Figure 1G). The instantaneous coefficient of friction (COF) was recorded under a sliding distance of 5 mm using a stainless‐steel ball (6 mm diameter) at room temperature (25°C). To simulate clinically relevant cartilage‐repair conditions, the applied load was fixed at 5 N, the sliding frequency was set to 1 Hz [48, 49], and physiological saline was used as the lubricant. As the sliding progressed, the COF of PSG‐Mg_c_ gradually stabilized, reaching an average value of ∼0.1, which is within or even lower than the reported range for native articular cartilage (0.05‐0.3) [50]. These results demonstrate that PSG‐Mg_c_ provides excellent lubrication and anti‐friction capability, which is critical for protecting newly regenerated cartilage under mechanical loading. Mechanistically, the cartilage‐like lubrication behavior arises from the synergistic effects of hydration‐layer formation, elastic recovery, and ion‐mediated interfacial interactions. The abundant hydrophilic groups and ionic crosslinks of PVA and SA promote the formation of stable hydration shells and high‐water retention, while the flexible Acy‐Gel segments enhance surface compliance under shear. Together, these features replicate the boundary‐lubrication mechanism of native cartilage and enable sustained low‐friction sliding during joint motion.

The structural evolution of PSG‐Mg_c_ hydrogel was investigated using the in situ small‐angle X‐ray scattering (SAXS) (Figure 1H). SAXS enables real‐time monitoring of nanoscale rearrangement within hydrated polymer networks, as variations in scattering intensity directly reflect changes in domain spacing, orientation, and network anisotropy [51, 52]. By coupling SAXS with tensile deformation, we tracked how polymer chains, crystalline domains, and ion‐mediated network structures reorganize during stretching, thereby elucidating the microscopic basis of hydrogel toughening.

The 2D SAXS patterns collected at tensile strains of 0%, 100%, and 200% are shown in Figure 1H a–c. The toughening behavior is mainly attributed to the PVA crystalline domains formed during freeze‐thaw processing together with the interpenetrating SA/Acy‐Gel network. According to conventional definitions [53, 54], the meridional and equatorial directions correspond to scattering parallel and perpendicular to the stretching direction, respectively. In the relaxed state, PSG‐Mg_c_ exhibited an isotropic scattering pattern, indicating randomly oriented lamellar crystalline domains (Figure 1Ha). Upon stretching to 100% and 200% strain (Figure 1Hb,c), scattering intensity progressively increased along the meridional direction (as indicated by the arrow) while decreasing along the equatorial direction, forming distinct streak‐like features. This anisotropic evolution arises from changes in inter‐fibrillar spacing and domain alignment rather than microcrack formation, reflecting a transition from randomly distributed lamellae to oriented fibrous crystalline structures.

The azimuthally integrated 1D SAXS profiles at tensile strains of 0%, 40%, 80%, 100%, and 200% are summarized in Figure 1I. All curves gradually flattened in the low‐Q region (5‐20 (×10^−3^ Å^−^^1^)) with increasing strain, indicating enhanced nanoscale homogeneity due to network dilation. Meanwhile, the overall scattering intensity decreased, consistent with a reduced density of scattering centers and the development of anisotropy. In the high‐Q regime (Q > 40 (×10^−3^ Å^−^^1^)), all samples displayed low and nearly overlapping intensities, suggesting that the local conformation of polymer chains and solvent interfaces remained unchanged during deformation [55, 56]. Together, these results demonstrate that mechanical loading primarily induces reorganization of the mesoscale network architecture while preserving local chain structures, thereby establishing a stable and anisotropic framework that facilitates the migration and regulation of functional cations (e.g., Mg^2+^) within the PSG‐Mg_c_ hydrogel.

### 2.2 Electrochemical Properties of Piezo‐Mimetic Ionic Hydrogels

Given that electrical stimulation is known to recapitulate endogenous microcurrents that regulate chondrocyte differentiation, matrix synthesis, and cartilage regeneration, we next examined whether the mechanically robust PSG‐Mg_c_ hydrogel could convert compressive deformation into effective electrical signals. To visualize the underlying piezo‐mimetic ionic mechanism, COMSOL finite‐element simulations were performed to analyze stress distribution and electrical potential generation within the hydrogel under compression (Figure 1J). As the applied load deformed the hydrogel network (Figure 1K,L), a spatially distributed electrical potential was generated (Figure 1M). This effect originates from deformation‐induced migration of mobile ions, which leads to transient charge separation between cations and anions, thereby producing a measurable potential difference that functionally mimics a piezoelectric response.

To experimentally validate this behavior, PSG_c_ and PSG‐Mg_c_ hydrogels were subjected to cyclic compressive loading using a custom‐built stepwise loading device (Figure 1N). Both hydrogels exhibited periodic current responses synchronized with mechanical loading. However, PSG_c_, which was equilibrated only in PBS, generated only weak electrical signals due to the limited availability of mobile ions. In contrast, PSG‐Mg_c_ produced a markedly higher and more stable output current of approximately 20 µA. This enhancement arises from the robust polymeric network formed by the freeze‐thaw induced crystalline domains of PVA interpenetrated with SA/Acy‐Gel chains, where their negatively charged carboxylate groups effectively attract and coordinate mobile cations. In addition, the incorporation of Acy‐Gel introduces flexible chain segments that facilitate network deformation and ion mobility, collectively contributing to the microstructural evolution revealed in the SAXS patterns shown in Figure 1H,I. Moreover, the introduction of Mg^2+^ enhances the output current of the PSG‐Mg_c_ hydrogel primarily due to its highly dynamic ionic coordination, which facilitates ion mobility within the network. It comes that Mg^2+^ possesses a smaller ionic radius and a more strongly bound hydration shell, which makes its coordination with carboxyl groups less stable and predominantly monodentate [57, 58]. Consequently, the PSG‐Mg_c_ hydrogel, with its superior mechanical integrity and structural stability, enabled uniform stress transmission during deformation, thereby promoting efficient ion movement and enhancing the generated electrical signal.

Finally, to establish a direct mechanical‐electrical coupling relationship, synchronized force‐current measurements were performed on PSG‐Mg_c_ under compressive loads of 5, 10, and 15 N (Figure 1O). The output current increased proportionally with applied stress, and the electrical signals were precisely synchronized with the loading cycles. This linear, reproducible, and stable response confirms that the PSG‐Mg_c_ hydrogel effectively converts mechanical stimuli into electrical outputs through ion migration, thereby functioning as a reliable piezo‐mimetic ionic hydrogel scaffold for cartilage repair applications.

### 2.3 Sensing Properties of Piezo‐Mimetic Ionic Hydrogels

As shown in Figure 1O, the output current of the hydrogel exhibited a slight temporal delay following mechanical loading, reflecting the intrinsic ion‐transport kinetics of the network. Because charge conduction in this system is dominated by ion migration, this response behavior closely resembles the signal transduction mode of native biological tissues, where ionic currents mediate mechanical and biochemical signaling.

To quantify the sensing capability of the hydrogels, we next evaluated their strain sensitivity. As illustrated in Figure 1P and Figure S11A, PSG‐Mg_c_ displayed higher strain‐dependent sensitivity and superior dynamic electromechanical performance compared with PSG_c_. The relative resistance of PSG‐Mg_c_ changed consistently with applied strain, indicating stable and reliable sensing behavior.

Response time is another critical parameter that defines how rapidly mechanical stimuli can be converted into electrical signals in a hydrogel. As shown in Figure 1Q and Figure S11B, the response times of PSG_c_ and PSG‐Mg_c_ were 50 and 45 ms, respectively. For reference, human skin exhibits a strain response time of approximately 15 ms [59], whereas articular cartilage, due to its high stiffness and fluid‐dependent viscoelasticity, shows much slower recovery, with patellar cartilage deformation persisting for hours after exercise [60]. These results demonstrate that PSG‐Mg_c_ achieves rapid and synchronized signal transduction, with resistance changes closely tracking applied strain in real time. Moreover, repeated loading‐unloading cycles under constant strain confirmed its excellent sensing stability and reversibility, highlighting its potential as a bioinspired mechanosensory scaffold for cartilage repair.

On the other hand, the PSG‐Mg_c_ hydrogel exhibits frequency‐dependent piezo‐mimetic electrical responses, where the output signal closely follows the applied mechanical frequency. As shown in Figure S12, increasing the loading frequency from 1 to 5 Hz results in a higher oscillation frequency of the current signal, accompanied by a slight reduction in amplitude. This behavior reflects the intrinsic ion transport kinetics within the hydrated network, where sufficient ion redistribution at lower frequencies leads to higher signal output, while limited migration at higher frequencies slightly attenuates the response, highlighting the dynamic and adaptable ion‐conductive nature of the hydrogel system. This suggests that the material is capable of adapting to dynamic joint motion conditions in vivo.

### 2.4 Piezo‐Mimetic Ionic Hydrogels Promote Chondrogenic Differentiation in Vitro

We hypothesized that the piezo‐mimetic ionic hydrogels could generate endogenous bioelectricity‐like ionic signals in response to mechanical stimulation, thereby promoting the chondrogenic differentiation of stem cells. Bone marrow‐derived stem cells (BMSCs), owing to their strong multipotency and physiological relevance to articular tissues, were selected as the in vitro model.

**FIGURE 2:**
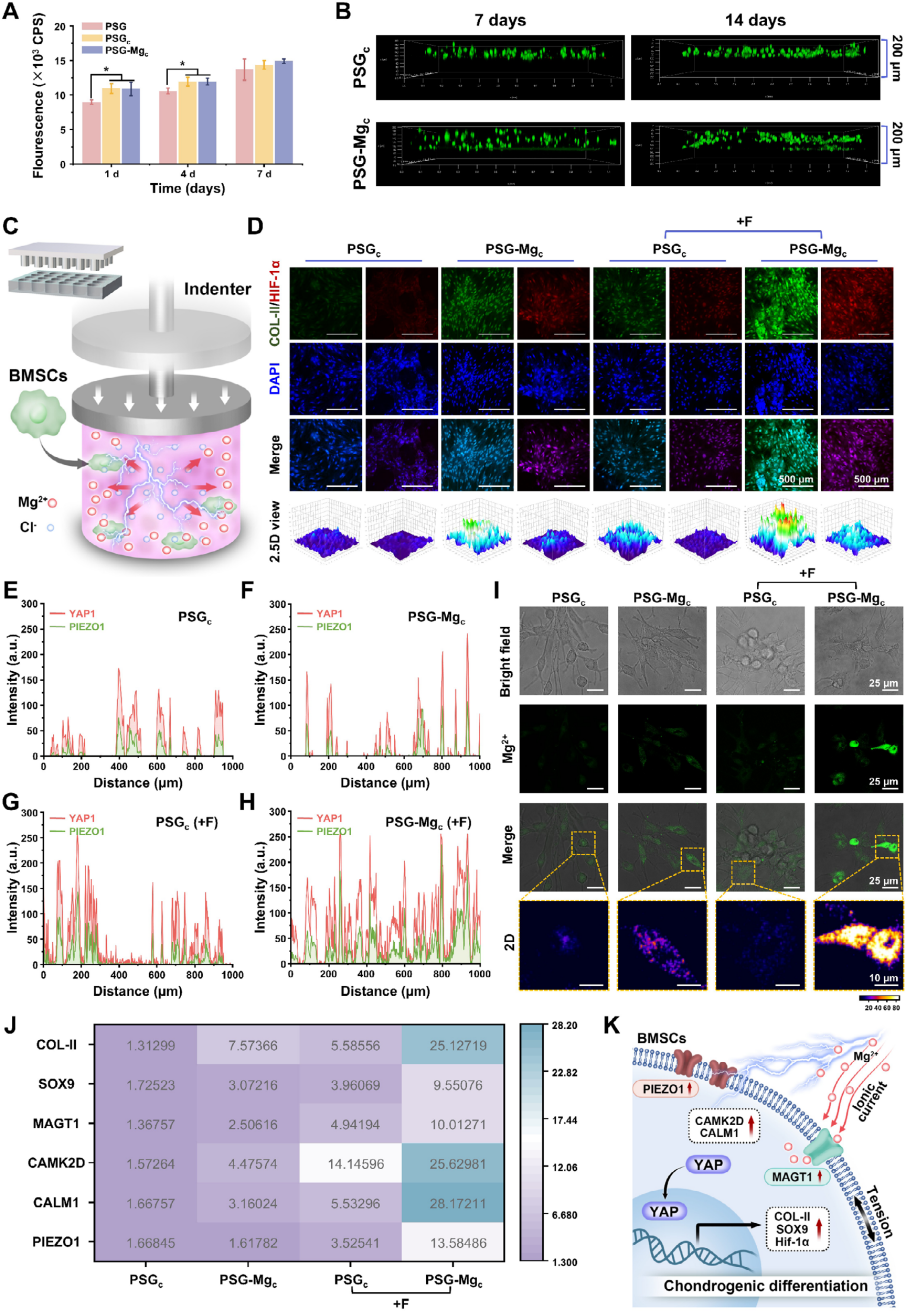
Piezo‐mimetic ionic hydrogels promote mechano‐electrical coupling chondrogenic differentiation of BMSCs under compressive stimulation. (A) Cytocompatibility of PSG, PSG_c_, and PSG‐Mg_c_ hydrogels evaluated by CCK‐8 assay. (B) Evaluation of the migration ability of BMSCs into the interior of PSG_c_ and PSG‐Mg_c_ hydrogels. (C) Schematic of the in vitro mechanical stimulation system applied to piezo‐mimetic ionic hydrogels. (D) CLSM images showing the immunofluorescence staining of COL‐II and HIF‐1α in BMSCs cultured on compressed (PSG_c_(+F) and PSG‐Mg_c_(+F)) and non‐compressed (PSG_c_ and PSG‐Mg_c_) hydrogels, after 14 days of chondrogenic induction. (E–H) Co‐localization analysis of YAP1 and PIEZO1 in BMSCs cultured on PSG_c_ and PSG‐Mg_c_ hydrogels under mechanical and non‐mechanical conditions. (I) CLSM images showing the intracellular Mg^2+^ visualization in BMSCs cultured on PSG_c_ and PSG‐Mg_c_ with and without mechanical stimulation. (J) Quantification of related genes by qRT‐PCR, including cartilage matrix synthesis‐related genes (COL‐II and SOX9); Mg^2+^ transporter protein‐related gene (MAGT1); calmodulin‐related genes (CAMK2D and CALM1); ion‐channel‐related gene (PIEZO1). (K) Proposed mechano‐electrical coupling mechanism by which the piezo‐mimetic ionic hydrogel regulates BMSC chondrogenic differentiation. Results are presented as mean ± SD (*n* ≥ 4). ^*^ *p* < 0.05.

Cell viability and proliferation were first assessed using quantitative assays (Figure 2A) and live/dead staining (Figure S13). Both PSG_c_ and PSG‐Mg_c_ exhibited no cytotoxicity and instead slightly promoted BMSCs proliferation. On days 1 and 4, these two groups showed significantly higher viability than PSG. After 7 days, BMSCs displayed well‐spread morphologies on all hydrogels (Figure S13). Notably, PSG‐Mg_c_ promoted more abundant extracellular matrix deposition, accompanied by the lowest dead‐cell ratio, highlighting the positive regulatory effect of bioactive Mg^2+^ ions.

To ensure effective mechano‐electrical regulation, BMSCs must migrate into the hydrogel interior. Cells were seeded onto PSG_c_ and PSG‐Mg_c_ and cultured for 14 days, followed by 3D fluorescence tomography. Confocal laser scanning microscopy (CLSM) images showed that cells in PSG‐Mg_c_ penetrated significantly deeper than those in PSG_c_, indicating that Mg^2+^ incorporation facilitates cell migration within the porous network (Figure 2B).

A custom in vitro stimulation system was established to apply defined cyclic compression (Figure 2C). After stimulation, expression of chondrogenic markers COL‐II and HIF‐1α was evaluated (Figure 2D). Mechanical loading (the two groups labeled with +F) markedly enhanced chondrogenic differentiation in both hydrogels; however, PSG‐Mg_c_(+F) showed the strongest effect, consistent with its higher piezo‐mimetic ionic output. Even without stimulation, PSG‐Mg_c_ promoted baseline chondrogenic marker expression due to Mg^2+^ release. These findings were further confirmed by semi‐quantitative fluorescence analysis (Figure S14).

YAP is a key mechanotransducer that responds to mechanical deformation by transmitting physical cues from the cytoskeleton to the nucleus, thereby initiating downstream signaling cascades [61, 62]. PEIZO1 is a mechanosensitive ion channel whose activation can be facilitated by membrane depolarization and enhanced local ionic gradients generated during mechanical deformation. The piezo‐mimetic ionic potentials and ion currents produced by the hydrogel effectively lower the activation threshold of PIEZO1, thereby amplifying mechanotransductive signaling [63, 64]. To elucidate the mechano‐electrical coupling mechanism, we performed co‐localization analysis of YAP1 and PIEZO1 (Figure 2E–H; Figure S15). Under cyclic mechanical, both PSG_c_(+F) and PSG‐Mg_c_(+F) groups exhibited increased YAP1 expression compared with their unloaded counterparts (PSG_c_ and PSG‐Mg_c_) (Figure S15), confirming activation of mechanical signaling. Notably, PSG‐Mg_c_(+F), which generated stronger piezo‐mimetic ionic signals due to enhanced ion migration, displayed a concomitant ∼2‐fold upregulation of PIEZO1 alongside YAP1 activation (Figure 2H). These results indicate that mechanical deformation first activates YAP1, which in turn facilitates the opening of PIEZO1 channels. The resulting ion influx, together with deformation‐induced piezo‐mimetic ionic potentials, synergistically establishes localized bioelectrical microenvironments that reinforce intracellular signaling. Through this coordinated YAP‐mediated mechanotransduction and PIEZO1‐dependent ion transport, the piezo‐mimetic ionic hydrogel effectively promotes chondrogenic differentiation and extracellular matrix synthesis in BMSCs, demonstrating a synergistic mechano‐electrical coupling mechanism.

Mg^2+^ serves as a critical intracellular regulator of cellular bioenergetics, modulating stem‐cell metabolism by elevating ATP production, suppressing cyclin expression, and upregulating the chondrogenesis‐related transcription factor SOX9, thereby preserving the chondrogenic differentiation potential of stem cells [65, 66]. To visualize intracellular Mg^2+^ dynamics, Mag‐Fluo‐4 AM staining was used to monitor Mg^2+^ distribution in BMSCs cultured on piezo‐mimetic ionic hydrogels under simulated mechanical stimulation. As depicted in Figure 2I, even in the absence of external loading, cells on PSG‐Mg_c_ exhibited moderately enhanced intracellular Mg^2+^ levels relative to PSG_c_, reflecting the sustained Mg^2+^ release from the scaffold. Upon mechanical stimulation, this effect was markedly amplified in the PSG‐Mg_c_(+F) group, as evidenced by significantly stronger Mg^2+^‐dependent fluorescence, indicating mechano‐electrically enhanced Mg^2+^ uptake. These results demonstrate that, in addition to promoting Ca^2+^ influx, activation of the mechanosensitive PIEZO1 channel under piezo‐mimetic ionic stimulation also induces dynamic oscillations in intracellular Mg^2+^, providing an additional bioenergetic and transcriptional regulatory axis that supports chondrogenic differentiation.

Finally, the expression of mechanosensitive and calmodulin‐related genes, including CAMK2D, CALM1, and PIEZO1, was quantified to verify activation of downstream mechano‐electrical signaling pathways. As shown in Figure 2J, mechanical loading significantly upregulated all three genes in both PSG_c_(+F) and PSG‐Mg_c_(+F) groups compared with their unstimulated counterparts. Notably, PSG‐Mg_c_(+F) elicited a much stronger transcriptional response, with several genes expressed at levels approximately 2∼5‐fold higher than those observed in PSG_c_(+F).

In parallel, MAGT1, a key regulator of intracellular Mg^2+^ transport and homeostasis, was also markedly elevated under mechanical stimulation, indicating enhanced Mg^2+^ metabolic activity. The sustained release of Mg^2+^ from PSG‐Mg_c_ further amplified MAGT1 expression, which subsequently promoted the expression of cartilage‐associated genes COL‐II and SOX9 and strengthened the chondrogenic differentiation of BMSCs (Figure 2K). Together, these findings demonstrate that cells residing within the PSG‐Mg_c_ scaffold directly sense deformation‐induced ion transport and piezo‐mimetic ionic electrical signals, which modulate membrane potential and activate a cascade of mechanosensitive and Mg^2+^‐dependent pathways that collectively drive chondrogenic commitment.

### 2.5 Cartilage Defect Repair in Rabbit Knee Joints Under Distinct Mechanical Loading Conditions

**FIGURE 3:**
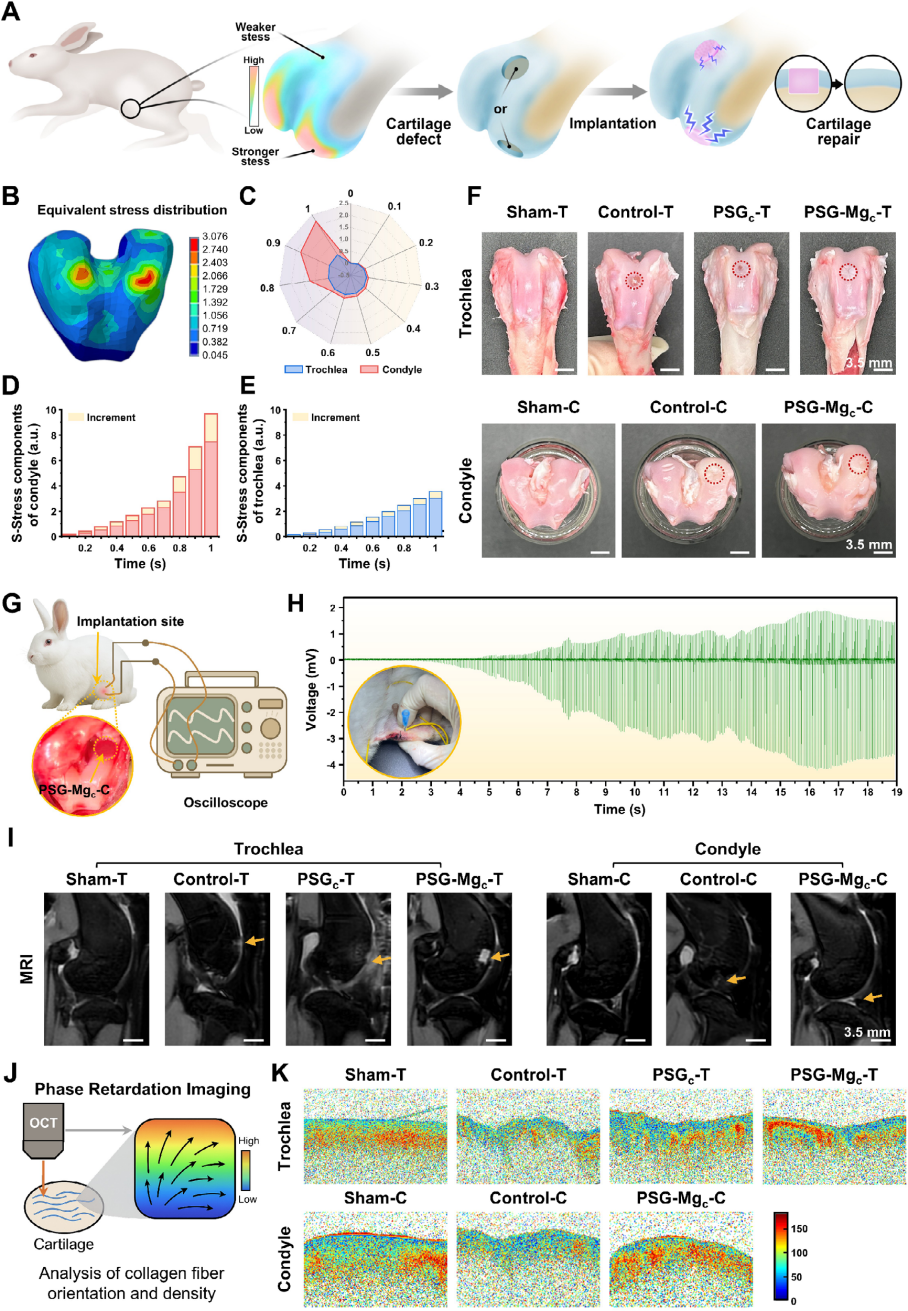
In vivo evaluation of cartilage regeneration under distinct mechanical loading conditions using a piezo‐mimetic ionic hydrogel. (A) Schematic representation of two anatomically distinct rabbit cartilage defect models established in the trochlea groove (low mechanical loading) and the femoral condyle (high mechanical loading), designed to systematically elucidate the impact of mechanical force magnitude on the performance of the piezo‐mimetic ionic hydrogel. (B,C) FEA‐simulated stress distribution contour profile at the trochlea (T) and femoral condyle (C) regions. (D,E) Quantitative analysis of cumulative stress and mechanical force increment at the trochlea and femoral condyle regions over time. (F) Representative macroscopic images of femoral trochlea (top) and medial femoral condyle (bottom) harvested from Sham, Control, PSG_c_, and PSG‐Mg_c_ treatment groups. Red dashed circles indicate defect regions, highlighting the extent of defect filling and surface restoration after implantation under distinct mechanical loading environments. (G) Schematic illustration of the force‐dependent electrical signal response of the piezo‐mimetic ionic hydrogel implanted into an in situ femoral condyle cartilage defect in a rabbit, enabling in vivo monitoring. (H) Representative in vivo recordings of force‐induced electrical signal from the piezo‐mimetic ionic hydrogel implanted into an in situ femoral‐condyle cartilage defect in a rabbit. (I) MRI assessment of cartilage repair outcomes. (J) Schematic illustration of the heterogeneous structure of regenerated neo‐cartilage revealed by phase retardation analysis. (K) Slope‐based analysis of phase retardation. Results are presented as mean ± SD (*n* ≥ 4).

To further verify the therapeutic efficacy of the piezo‐mimetic ionic hydrogel with Mg^2+^‐supply in promoting in vivo cartilage regeneration, two anatomically distinct cartilage defect models were established at the trochlear groove and the femoral condyle (Figure 3A). The trochlear cartilage is subjected to relatively mild mechanical stimulation from the patella, whereas the femoral condyle experiences substantially higher mechanical loading during joint motion. Finite element analysis (FEA) was therefore performed to quantify the loading disparity between these two regions. The resulting stress‐distribution maps confirmed that the femoral condyle was exposed to significantly higher mechanical stress than the trochlear region (Figure 3B,C). Moreover, the magnitude and accumulation of stress at the femoral condyle increased markedly with prolonged loading time (Figure 3D,E).

To assess the in vivo piezoelectric performance of the hydrogel under physiological loading, PSG‐Mg_c_ hydrogels were implanted into φ = 3.5 mm full‐thickness cartilage defects in the femoral condyles of rabbit knees (designated as PSG‐Mg_c_‐C group). Untreated femoral condyle defects and sham‐operated condyles were designated as the Control‐C and Sham‐C groups, respectively. To delineate the specific contribution of the piezoelectric effect, PSG‐Mg_c_ was also implanted into the low‐load trochlear defects (PSG‐Mg_c_‐T group). To isolate the role of Mg^2+^ release, PSG_c_ without Mg^2+^ was implanted into the trochlea defects (PSG_c_‐T group). In addition, untreated and sham‐operated trochlea defects were defined as Control‐T and Sham‐T groups, respectively.

After 8 weeks of implantation at both defect sites, macroscopic evaluation of the distal femur revealed that the Control‐T group exhibited persistent defects at the trochlea site, reflecting the limited intrinsic repair capacity of cartilage under low mechanical stimulation (Figure 3F). The PSG_c_‐T group showed partial defect filling, indicating that the hydrogel scaffold provided mechanical buffering and reduced friction‐induced damage. Notably, both PSG‐Mg_c_‐T and PSG‐Mg_c_‐C groups demonstrated substantial cartilage regeneration. In the trochlea region, Mg^2+^ release promoted cellular proliferation and matrix deposition, resulting in broad defect coverage by fibrocartilage, although fissures remained in the central zone. In contrast, in the high‐stress femoral condyle, the PSG‐Mg_c_‐C group exhibited smooth, continuous, and well‐integrated neocartilage that closely resembled native tissue in the Sham‐C group.

Real‐time electrical monitoring further confirmed that the PSG‐Mg_c_‐C hydrogel group generated detectable electrical signals in response to joint loading, with the output voltage displaying periodic fluctuations under physiological motion (Figure 3G,H). Besides, magnetic resonance imaging (MRI) performed at 8 weeks post‐implantation revealed residual fluid accumulation in the Control‐T and Control‐C groups (Figure 3I). The PSG_c_‐T group exhibited partial filling but with localized thickening and small unfilled gaps. The PSG‐Mg_c_‐T group showed markedly improved repair, although the regenerated cartilage remained discontinuous, consistent with macroscopic findings. In contrast, the PSG‐Mg_c_‐C group achieved uniform and complete defect coverage without excessive cartilage overgrowth.

To evaluate the collagen organization within the regenerated cartilage, optical coherence tomography (OCT)‐based phase retardation analysis was performed (Figure 3J). OCT analyze enables non‐destructive, depth‐resolved evaluation of cartilage by quantifying collagen birefringence, thereby reflecting the orientation and zonal organization of collagen fibers [67]. Compared with conventional histology, OCT provides real‐time, label‐free imaging across the full cartilage thickness and allows quantitative assessment of collagen alignment and structural integrity. These advantages make OCT particularly suitable for evaluating the restoration of depth‐dependent collagen architecture during cartilage regeneration. As shown in Figure 3K, the Sham‐T and Sham‐C groups displayed a progressive increase in birefringence from the superficial to deep zones, indicative of native zonal architecture. Conversely, the Control‐T and PSG_c_‐T groups showed irregular surfaces and disorganized collagen alignment. Although the PSG‐Mg_c_‐T group presented a smoother surface, structural discontinuities were still observed centrally. In the femoral condyle model, the Control‐C group exhibited severe disruption of deep‐zone collagen. In contrast, the PSG‐Mg_c_‐C group showed well‐organized collagen fibers with an architecture closely resembling native cartilage. Furthermore, the PSG‐Mg_c_‐C group exhibited distinct concentric lamellar patterns and stable depth‐dependent collagen organization, comparable to the Sham‐C group (Figure S16).

**FIGURE 4:**
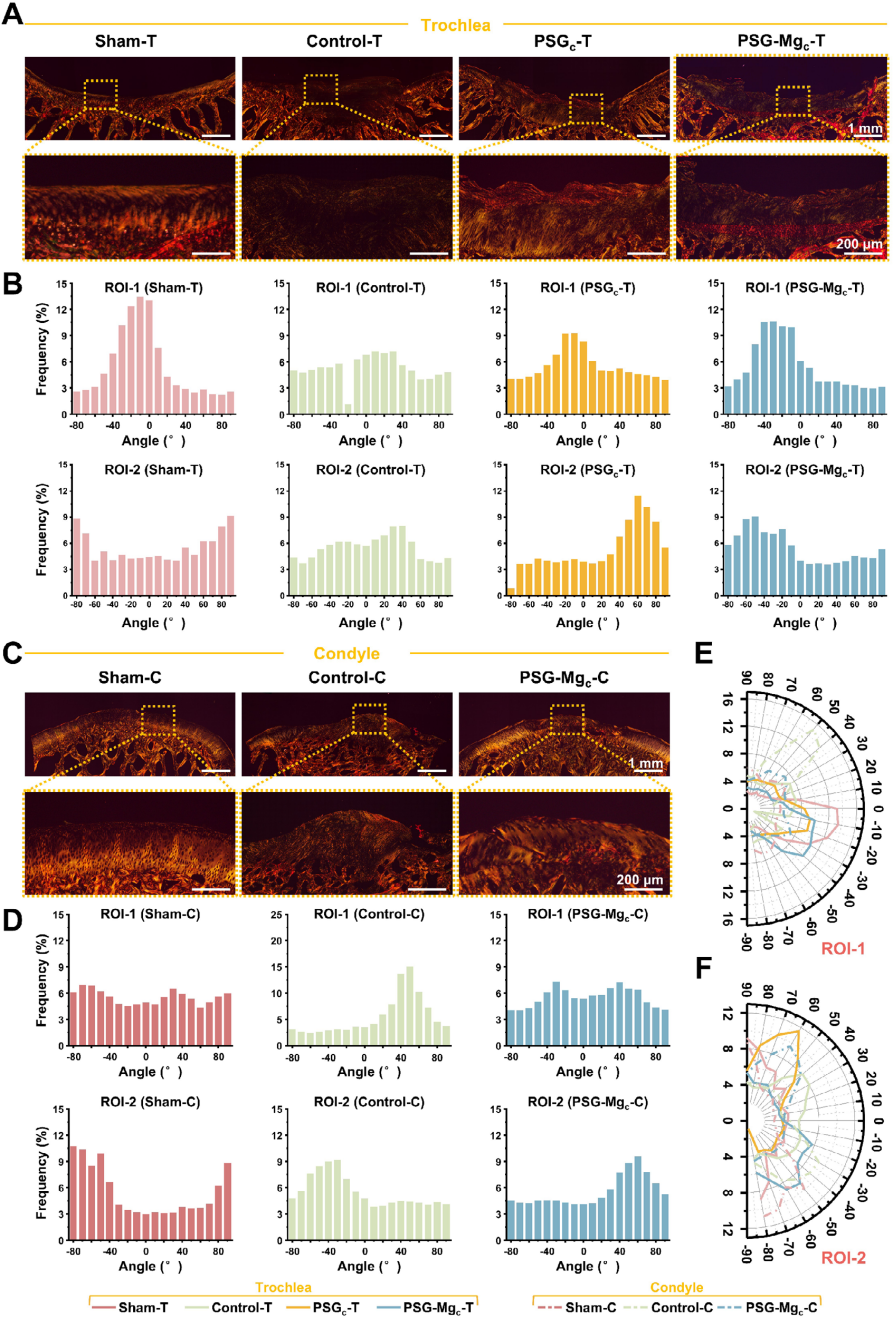
Picrosirius red staining of collagen fibers in cartilage tissues from rabbit knee joints at 8 weeks post‐operation. (A,C) Representative histological sections from the trochlea and condyle regions imaged under polarized light microscopy; lower panels show the higher‐magnification views of the boxed areas. (B,D) Orientation and dispersion of collagen fibers in the ROI‐1 and ROI‐2 from the respective regions. (E,F) Polar coordinate analysis of collagen fiber orientation and dispersion across groups. Fibers parallel to the articular surface are defined as 0°, and those perpendicular as 90°. Results are presented as mean ± SD (*n* ≥ 4).

Furthermore, Sirius Red staining was also performed to assess collagen orientation in regenerated cartilage at the trochlear and femoral condyle sites. The Sham‐T and Sham‐C groups exhibited well‐organized depth‐dependent collagen alignment typical of native cartilage, while the Control‐T and Control‐C groups showed disordered and fragmented fibers, consistent with poor structural repair observed by OCT (Figure 4A–D). The PSG_c_‐T group demonstrated partial collagen recovery but retained irregular orientation. In contrast, the PSG‐Mg_c_‐T group displayed uniformly aligned fibers predominantly oriented along the loading direction, with narrower angular dispersion and higher directional coherence. In the high‐load femoral condyle model, collagen alignment in the PSG‐Mg_c_‐C group has closely resembled that of native cartilage. The corresponding polar plots indicate that the synergistic effects of mechanical loading and piezoelectric signaling significantly enhanced chondrocyte‐mediated matrix remodeling, thereby promoting structurally and functionally superior cartilage regeneration (Figure 4E,F).

**FIGURE 5:**
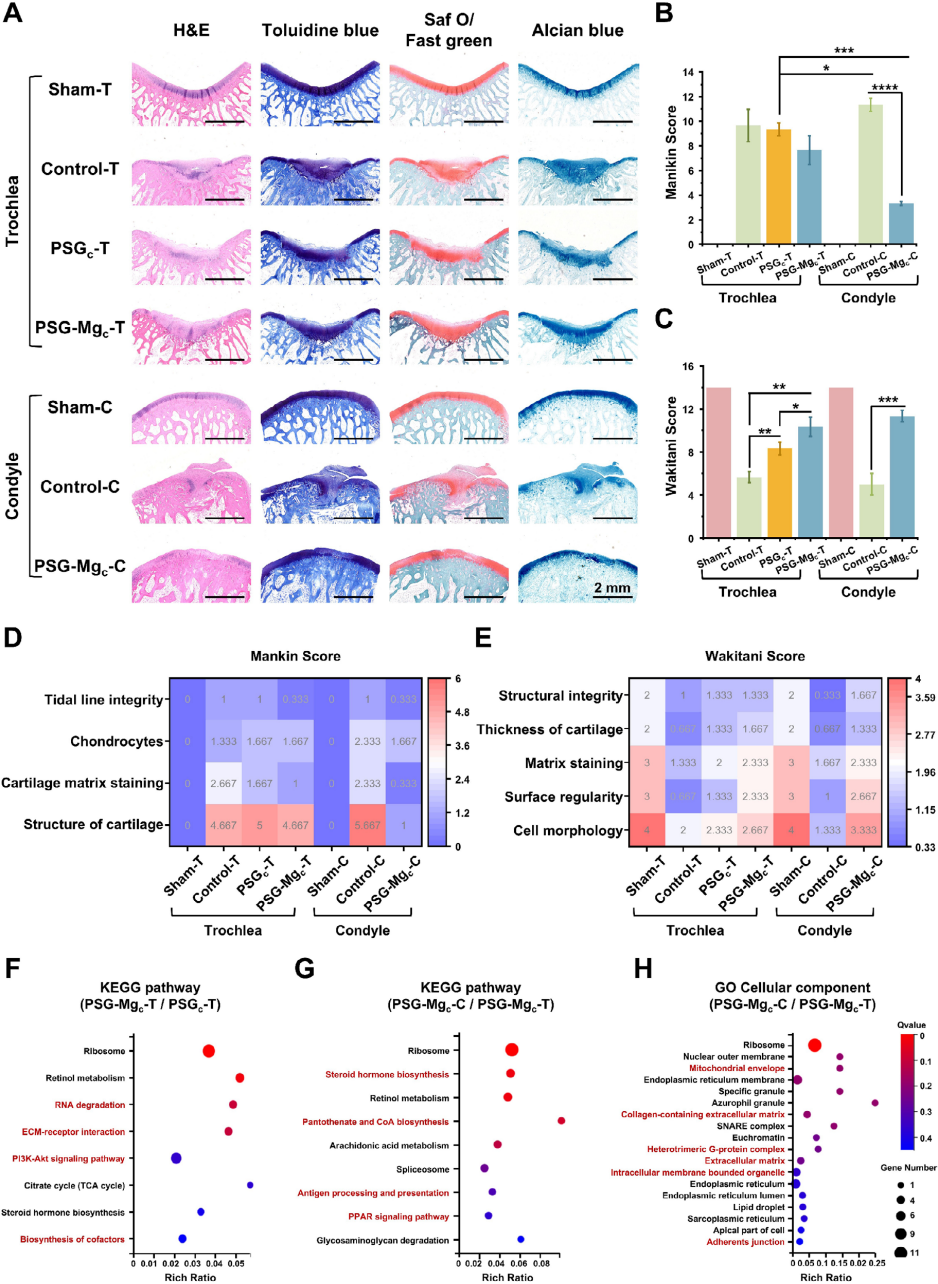
Biological evaluation of piezo‐mimetic ionic hydrogels implanted in the femoral condyle and trochlea regions of rabbit knee joints after 8 weeks of cartilage regeneration. (A) Representative images of the Hematoxylin and Eosin (H&E), Toluidine blue, Saf O/Fast green, and Alcian blue staining at 8 weeks post‐surgery. (B,C) The Manikin score and Wakitani score are analyzed and assigned accordingly. (D,E) Detailed criteria of Mankin score and Wakitani score for evaluating regenerated cartilage at the trochlea region and femoral condyle region. (The Mankin score includes assessments of Tidal line integrity, Chondrocytes, Cartilage matrix staining, and Structure of cartilage; The Wakitani score includes evaluations of Structural integrity, Thickness of cartilage, Matrix staining, Surface regularity, and Cellular morphology.) (F–H) Transcriptomic analysis of in situ cartilage regeneration promoted by the piezo‐mimetic ionic hydrogels: KEGG pathway enrichment analysis comparing the PSG‐Mg_c_‐T and PSG_c_‐T groups at the trochlea region (F); KEGG pathway enrichment analysis comparing the PSG‐Mg_c_‐C group at the femoral condyle region and the PSG‐Mg_c_‐T group at the trochlea region (G); GO cellular component analysis comparing the PSG‐Mg_c_‐C group at the femoral condyle region and the PSG‐Mg_c_‐T group at the trochlea region (H). Results are presented as mean ± SD (*n* ≥ 3). ^*^ *p* < 0.05, ^**^ *p* < 0.01, ^***^ *p* < 0.001, ^****^ *p* < 0.0001.

Histological staining was performed to evaluate cartilage regeneration at both the trochlear and femoral condyle defect sites (Figure 5A). The Sham‐T and Sham‐C groups exhibited continuous hyaline cartilage with smooth surface morphology and intact subchondral bone, characteristic of native tissue architecture. In contrast, the Control‐T and Control‐C groups showed incomplete defect filling, irregular surfaces, and weak staining, suggesting poor cartilage regeneration. The PSG_c_‐T group showed partial matrix deposition with uneven toluidine blue and Safranin O/Fast green (Saf O/Fast green) staining, indicating limited proteoglycan formation. Although the PSG‐Mg_c_‐T group exhibited more continuous neo‐cartilage formation and greater collagen deposition than the PSG_c_‐T group, the regenerated cartilage layer was markedly thickened and predominantly composed of fibrocartilaginous tissue. In contrast, the PSG‐Mg_c_‐C group exhibited uniform and intense staining across all histological assays, with well‐integrated cartilage‐like tissue and abundant GAG deposition, closely resembling native cartilage structure. Consistently, the PSG‐Mg_c_‐C group exhibited the lowest Mankin score and the highest Wakitani score among all treatment groups, excluding the Sham groups (Figure 5B–E), particularly in the mechanically loaded femoral condyle model. These results highlight the beneficial role of piezo‐mimetic ionic stimulation coupled with Mg^2+^ release in promoting cartilage regeneration.

To elucidate the molecular mechanisms underlying these regenerative outcomes, transcriptomic analysis was performed on regenerated cartilage tissues. Trochlear defect experiencing minimal mechanical loading were defined as the non‐stimulated condition (PSG_c_‐T and PSG‐Mg_c_‐T groups). Compared with PSG_c_‐T, the PSG‐Mg_c_‐T group exhibited significant upregulation of genes associated with ECM‐receptor interaction and the PI3K‐Akt signaling pathway, indicating that Mg^2+^ release enhanced ECM synthesis and cell‐matrix interactions (Figure 5F). Femoral condyle defects subjected to higher mechanical loading were defined as the mechanically stimulated condition (PSG‐Mg_c_‐C). Relative to PSG‐Mgc‐T, KEGG pathway analysis revealed that PSG‐Mg_c_‐C upregulated genes involved in cellular metabolism, including hormone signaling, enzymatic processes, and antigen processing (Figure 5G). GO enrichment analysis further identified pathways related to collagen‐containing ECM and adherent junctions, suggesting that mechanical stimulation reinforced cellular adhesion and matrix organization (Figure 5H). In addition, GSEA results demonstrated that the PSG‐Mg_c_‐C group enriched genes associated with the PPAR signaling pathway (Figure S17). Given the established role of PPAR pathway plays in regulating chondrocyte metabolism, mitochondrial function, redox balance, and extracellular matrix homeostasis under mechanical stress [68, 69], its upregulation in PSG‐Mg_c_‐C indicates that piezo‐mimetic ionic stimulation and Mg^2+^‐mediated biochemical cues synergistically activate metabolic programs that support chondrocyte differentiation and ECM remodeling. Overall, the combined analysis of KEGG and GO cellular functional process demonstrates that the integration of mechanical stimulation, piezo‐mimetic ionic signaling, and Mg^2+^‐mediated biochemical regulation in the PSG‐Mg_c_‐C group coordinately modulates cellular growth, metabolism, and differentiation, ultimately promoting cartilage regeneration and the formation of neo‐cartilage with native‐like structural and functional integrity (Figures S18 and S19).

Collectively, the PSG‐Mg_c_ hydrogel establishes a mechano‐responsive piezo‐mimetic ionic microenvironment, in which mechanical deformation induces asymmetric migration of anions and cations, generating localized charged potentials that actively regulate cellular behavior. This mechanically gated ionic signaling, together with the sustained release of bioactive Mg^2+^, synergistically reprograms stem‐cells metabolic activity, enhances mechanosensitive ion flux, and promotes chondrogenic differentiation through pathways such as PI3K‐Akt and PPAR, thereby improving cellular energy homeostasis, redox balance, and matrix remodeling. Consequently, extracellular matrix synthesis is markedly enhanced, the heterogeneous collagen architecture of native cartilage is restored, and achieved near‐complete regeneration of otherwise non‐self‐healing cartilage defects was achieved in vivo. These findings underscore the broad translational promise of piezo‐mimetic ionic hydrogels as next‐generation intelligent biomaterials capable of harnessing endogenous mechanical forces to direct tissue repair, offering a compelling platform for cartilage regeneration and other mechanically active tissues. Future work will focus on refining the spatiotemporal control of ionic signaling and advancing the system toward clinical translation for cartilage and other load‐bearing tissue repair.

## 3 Discussion

Cartilage regeneration remains a major clinical challenge due to the limited intrinsic repair capacity of articular cartilage and the difficulty of recapitulating its mechano‐electrical microenvironment. Native cartilage continuously experiences dynamic mechanical loading, which is transduced into ionic and electrical cues that regulate chondrocyte metabolism, matrix organization, and cell‐cell communication [70, 71]. However, most current biomaterial strategies focus primarily on structural support or biochemical supplementation [72, 73], failing to actively engage with these endogenous biophysical signals. Moreover, many existing electroactive scaffolds rely on externally applied electrical stimulation or electron‐based conduction mechanisms, which lack dynamic ion coupling and deviate from the ion‐mediated signaling processes of native cartilage. In this study, we introduce a piezo‐mimetic ionic hydrogel that directly couples physiological joint motion to localized ionic and electrical signaling, thereby enabling a dynamic and self‐sustaining regenerative microenvironment for cartilage repair.

The PSG‐Mg_c_ hydrogel integrates a Mg^2+^‐crosslinked sodium alginate network with a flexible, multicarboxylated Acy‐Gel matrix, yielding a hydrated, mechanically resilient scaffold that mimics key functional attributes of native cartilage. Beyond providing mechanical integrity and lubrication, this hybrid network functions as a mechano‐responsive ionic transducer. Under physiological loading, deformation of the charged polymer network induces asymmetric migration of cations and anions, generating localized ionic currents and transient bioelectric fields [74, 75]. This piezo‐mimetic ionic response converts otherwise dissipative mechanical energy into biologically meaningful signals, closely resembling ion‐based intercellular communication observed in healthy cartilage. Unlike conventional piezoelectric materials that rely on direction‐dependent polarization, this system generates electrical signals through deformation‐induced ion migration and is therefore not constrained by loading direction. As a result, it can respond to diverse mechanical stimuli, including compression, shear, and sliding, making it well suited for the multidirectional loading environment of articular cartilage.

A defining feature of this system lies in the dual and complementary coordination modes of Mg^2+^ provided by SA and Acy‐Gel, which together establish both mechanical integrity and dynamic ionic functionality. In the PSG‐Mg_c_ scaffold, SA forms a divalent cation–crosslinked network that confines Mg^2+^ ions within an eggshell‐like coordination architecture. This mode of binding enhances the load‐bearing capacity and mechanical stability of the hydrogel, allowing mechanical deformation to be efficiently translated into macroscopic stress resistance and structural reinforcement. By contrast, Acy‐Gel contains linearly distributed carboxyl groups that chelate Mg^2+^ through flexible and reversible ionic interactions, thereby generating a population of cations with higher mobility. These more freely mobile Mg^2+^ ions are crucial for deformation‐induced redistribution, sustained ion flux, and the generation of bioelectric signals under mechanical loading. The coexistence of mechanically stabilizing Mg^2+^ coordination by SA and dynamically responsive Mg^2+^ chelation by Acy‐Gel is essential for effective mechano‐electrical coupling. Together, these two coordination mechanisms enable spatially and temporally regulated Mg^2+^ signaling in response to joint motion, which synergizes with bioelectric cues to regulate cellular metabolism, reinforce mechanosensitive signaling pathways, and promote extracellular matrix remodeling during cartilage regeneration.

Conversely, from the perspective of ion coordination behavior and ionic mobility, Mg^2+^ and Ca^2+^ exhibit fundamentally different coordination characteristics. Mg^2+^ typically forms weaker and more dynamic coordination with carboxyl groups, often dominated by monodentate interactions and strong hydration shells, which facilitates faster ion exchange and higher mobility within hydrated polymer networks. In contrast, Ca^2+^ tends to form stronger and more stable ionic crosslinks, leading to a more rigid network but reduced ion mobility [76]. This difference has been widely reported in ionically crosslinked systems and is consistent with the observation that Mg^2+^‐containing hydrogels exhibit enhanced ion transport and dynamic behavior [77]. These properties enable Mg^2+^ to more effectively participate in deformation‐induced ion migration, thereby generating stronger piezo‐mimetic ionic signals. Meanwhile, the relatively weak Mg^2+^‐carboxyl interactions also allow gradual ion release in aqueous environments, providing sustained bioactive stimulation that regulates cellular metabolism and promotes chondrogenesis. Collectively, these processes act synergistically, where deformation‐driven ionic flux mimics endogenous electrochemical signaling to enhance mechanosensitive pathways, while sustained Mg^2^^+^ release supports extracellular matrix synthesis and tissue regeneration.

Importantly, this active signaling paradigm distinguishes the PSG‐Mg_c_ system from conventional inert or passively bioactive scaffolds. Rather than delivering static cues, the hydrogel continuously adapts its ionic and electrical outputs to the local mechanical environment, effectively closing the loop between joint motion and tissue regeneration. In vivo studies using anatomically distinct low‐ and high‐load cartilage defect models demonstrate that this mechano‐responsive behavior translates into robust cartilage regeneration with near‐native structural organization, even under challenging load‐bearing conditions. The depth‐dependent collagen arrangement and improved integration with surrounding tissue underscore the importance of mechanically gated ionic signaling in restoring functional cartilage architecture.

From a broader perspective, this work bridges a long‐standing gap between mechanical stimulation and biochemical regulation in cartilage repair. While external electrical stimulation and piezoelectric implants have shown promise [78, 79, 80], their reliance on rigid devices or exogenous power sources limits long‐term applicability and physiological relevance [81]. By contrast, the PSG‐Mg_c_ hydrogel operates as an intrinsically self‐powered system, harvesting endogenous joint motion to drive regenerative signaling without external hardware. This approach not only enhances translational potential but also aligns more closely with the principles of mechanobiology governing native tissue maintenance.

Several limitations and future directions warrant consideration. Although the present study demonstrates effective regeneration in rabbit models, further investigation is needed to elucidate the precise molecular pathways linking piezo‐mimetic ionic signaling to chondrogenic gene regulation, including the roles of mechanosensitive ion channels and downstream transcriptional networks. Long‐term studies in larger animal models will also be essential to evaluate durability, wear resistance, and integration under prolonged joint loading. Despite these remaining challenges, the design principles established in this work, including mechanical energy transduction, ionic conductivity, and dynamic biochemical regulation, are broadly applicable and may be extended to other mechanically active tissues such as the intervertebral disc, tendon, and osteochondral interfaces.

In summary, we present a piezo‐mimetic ionic hydrogel that transforms endogenous joint motion into coordinated ionic and electrical signals, driving stem cell chondrogenic differentiation and in situ cartilage regeneration. By shifting cartilage repair from passive mechanical support toward active mechano‐electrical coupling, this work establishes a generalizable framework for intelligent biomaterials that harness physiological motion to direct tissue regeneration.

## 4 Conclusion

In this study, we present a piezo‐mimetic ionic hydrogel that actively harnesses endogenous joint motion to drive cartilage regeneration through a synergistic mechano‐electrical mechanism. By integrating a Mg^2+^‐crosslinked sodium alginate network with an Acy‐Gel matrix, the PSG‐Mg_c_ hydrogel converts physiological mechanical loading into localized ionic currents, thereby generating dynamic bioelectric cues that act in concert with sustained Mg^2+^ release to regulate cellular metabolism, mechanosensitive signaling, and extracellular matrix remodeling. This piezo‐mimetic ionic coupling endows the hydrogel with key hallmarks of native cartilage, including mechanical resilience, hydration‐mediated lubrication, and an electro‐chemoactive microenvironment that supports depth‐dependent collagen organization and functional tissue regeneration in vivo.

Unlike conventional inert scaffolds that merely provide structural support, the PSG‐Mg_c_ system functions as a mechano‐responsive ionic transducer, enabling mechanically gated ion migration and self‐reinforcing regenerative signaling under joint motion. This dual‐mode regulation paradigm bridges the long‐standing gap between mechanical stimulation and biochemical modulation in cartilage repair, achieving near‐complete regeneration even in otherwise non‐healing defects. Overall, this work establishes a piezo‐mimetic design framework for intelligent biomaterials that integrate biomechanical sensing, ionic conductivity, and biochemical regulation, offering a broadly applicable strategy for regenerating cartilage and other mechanically active tissues.

## 5 Experimental Section

### 5.1 Materials

Polyvinyl alcohol (PVA, 1799), sodium alginate (SA), succinic anhydride, MgCl_2_, CaCl_2_, Tween 80, 1‐(3‐Dimethylaminopropyl)‐3‐ethylcarbodiimide hydrochloride (EDC), N‐Hydroxysuccinimide (NHS) were purchased from Aladdin Industrial Co., Ltd. (China). Gelatin (porcine skin, Bloom 250–300) (Gel), L‐proline, ITS, Triton X‐100, and TRIzol reagent were purchased from Sigma–Aldrich (USA). TGF‐β3 was purchased from R&D (USA). CCK‐8 kit and Calcein‐AM/PI were purchased from Dojindo (Japan). FITC‐phalloidine, Alcian blue staining kit, and 4,6‐diamidino‐2‐phenylindole (DAPI) were purchased from Solarbio (China). Mag‐Fluo‐4 AM was purchased from MKbio (China). The primary antibodies used for immunofluorescence and immunohistochemistry were purchased from Abcam (USA), and the secondary antibodies used were purchased from (ZSGB Bio, China). *α*‐Modified Eagle's Medium (*α*‐MEM), phosphate buffered saline (PBS), and penicillin‐streptomycin were purchased from Hyclone (USA). Fetal bovine serum (FBS) was purchased from Gibco (USA). ELISA kits were purchased from Blue gene (China). BCA protein assay kit was purchased from Thermofisher (USA). BSA was purchased from VWR Life Science (Ireland).

### 5.2 Synthesis and Characterizations of Acylated Gelatin (Acy‐Gel)

Gelatin (20 g) was dissolved in 100 mL of deionized water at 60°C under a water bath. The resulting solution was maintained at 40°C, and gradually added dropwise to adjust the pH to approximately 9.0 with NaOH (1 mol/L). Subsequently, succinic anhydride powder (2 g) was slowly introduced into the reaction system while maintaining the pH at ∼9.0, and the mixture was allowed to react for 30 min. The Acy‐Gel obtained was insoluble near its isoelectric point. Accordingly, the reaction mixture was adjusted with 0.001 mol/L HCl to pH 3.7–4.3, causing Acy‐Gel to precipitate. The precipitate was collected by filtration, repeatedly washed with distilled water, and dried for subsequent use.

The successful synthesis of Acy‐Gel was confirmed by Fourier transform infrared (FTIR) (Nicolet 6700, Thermo Fisher Scientific, USA). Spectra were recorded in the range of 500–2000 cm^−^^1^ and subsequently analyzed to verify the chemical structure of the modified gelatin. The degree of acylation (DA) of Acy‐Gel was determined using the formaldehyde titration method by quantifying the change in amino group content before and after modification. Briefly, 1 g native gelatin was dissolved in 150 mL deionized water at 45°C. The pH of the solution was adjusted to approximately 9.0 with 1 mol/L NaOH. Subsequently, a 25 mL formaldehyde (10%) solution was added, followed by readjustment of the pH to 9.0 using 0.02 mol/L NaOH. The volume of NaOH consumed at this stage was recorded as Y_1_. The same procedure was repeated for Acy‐Gel, and the corresponding NaOH consumption was recorded as Y_2_. To eliminate potential interference, a blank experiment was performed under identical conditions using deionized water instead of gelatin, and the NaOH consumption was recorded as Y_3_. The degree of acylation (a) was then calculated according to the following equation:

$$a\;=\frac{Y_{1}-Y_{2}}{Y_{1}-Y_{3}}\;\times 100\%$$

### 5.3 Preparation of the Piezo‐Mimetic Ionic Hydrogels

A 10 wt.% PVA solution was first prepared by dissolving the polymer under stirring at 90°C until complete solubilization. According to the formulation ratios listed in Table S1, three types of piezo‐mimetic ionic hydrogels were fabricated: PVA+SA (denoted PS), PVA+Acy‐Gel (denoted PG), and PVA+SA+Acy‐Gel (denoted PSG). To facilitate the migration of cells into the interior of the hydrogel, Tween 80 was introduced into the system as a porogen. Then, hydrogels were formed through freezing and thawing cycles. This process promoted the crystallization of the PVA network, yielding composite hydrogels consisting of linear alginate and acylated gelatin (Acy‐Gel) embedded within the physically crosslinked PVA.

The as‐prepared hydrogels were removed from the molds and washed with deionized water under magnetic stirring to eliminate residual Tween 80, thereby generating a porous structure. Finally, the hydrogels were immersed for at least 48 h in PBS, 0.5 mol/L CaCl_2_, or 0.5 mol/L MgCl_2_ solutions to achieve ionic equilibrium, resulting in piezo‐mimetic ionic hydrogel systems with distinct ionic‐crosslinking environments.

### 5.4 Covalent Crosslinking Treatment

To enhance the mechanical strength of the hydrogels and avoid the rapid loss of gelatin, the Acy‐Gel within the hydrogels was further chemically crosslinked. A solution containing NHS and EDC at a 1:2.5 ratio was prepared. PSG hydrogels were immersed in this solution at 4°C for 24 h. During this process, EDC activated the carboxyl groups of gelatin, which subsequently reacted with NHS to form amide bonds, thereby establishing a crosslinked gelatin network within the hydrogels. Subsequently, the hydrogels were rinsed with deionized water 3–5 times to remove unreacted reagents, equilibrated in PBS or 0.5 mol/L MgCl_2_ solution for 48 h to obtain PSG_c_ and PSG‐Mg_c_ hydrogels.

### 5.5 Characterizations of Hydrogels

The microstructures were investigated by using a scanning electron microscope (SEM, JEOL JSM‐7500F, Japan) at acceleration voltages of 15–20 kV, following the deposition of a thin gold layer on the surface via sputtering (Instrument E5600, Polaron, USA). The pore size of the freeze‐dried samples was analyzed and statistically calculated by Image‐Pro Plus (IPP) software, and the pore size distribution was plotted using Gaussian curves.

For the swelling‐degradation testing, all samples were lyophilized prior to measurement, and their initial dry weights were recorded as W_0_. The samples were then immersed in a fixed volume of PBS and incubated in a 37°C water bath. At predetermined time points, samples were removed, blotted with qualitative filter paper to eliminate excess surface water, and weighed to obtain W_t_. The swelling ratio (water absorption ratio) of each sample was calculated using the following equation:

$$\mathrm{Water}\;\mathrm{absorption}\;\mathrm{ratio}\;=\frac{W_{t}-W_{0}}{W_{0}}\;\times 100\%$$

### 5.6 Evaluation of Ca^2+^/Mg^2+^ Uptake and Release

PS, PG, and PSG hydrogels were immersed in 10 mL of pre‐prepared 0.5 mol/L CaCl_2_ or 0.5 mol/L MgCl_2_ solutions. The systems were stirred for 48 h to allow sufficient interaction between cations and the polymer networks. After that, the residual solutions were collected, and the concentrations of residual Ca^2+^ and Mg^2+^ in the solutions were measured by inductively coupled plasma optical emission spectrometry (ICP‐OES‐7500, Shimadzu, Japan). Based on the initial ion concentrations (C = 0.5 mol/L), the absorption capacity of each hydrogel group for Ca^2+^ and Mg^2+^ was calculated.

To investigate the ion release from these ion‐equilibrated hydrogels, they were transferred into 5 mL of deionized water, incubated at 37°C on a shaker (50 rpm) for 14 days. At predetermined time intervals, aliquots of the supernatant were collected, and the concentrations of Ca^2+^ and Mg^2+^ were quantified using ICP‐OES‐7500. The cumulative release profiles of Ca^2+^ and Mg^2+^ over 14 days were subsequently calculated.

### 5.7 Mechanical Performance Testing

For the tensile performance testing, the PS, PG, and PSG hydrogels equilibrated with Ca^2+^ and Mg^2+^ were molded into a dumbbell‐shaped geometry using a custom‐designed mold, with a width of 2 mm and a gauge length of 10 mm. The specimens were stretched at a constant elongation rate of 1 mm/min until fracture occurred at the midpoint of the sample, at which point the test was terminated.

For the compression and the cyclic compression tests, hydrogels were prepared with 10 mm in diameter and 5 mm in height. Mechanical testing was performed using a universal testing machine in compression mode at a constant rate of 1 mm/min, and the corresponding stress–strain curves were recorded for each sample group. On the other hand, hydrogels were subjected to cyclic compression test in an isostatic mode with a loading rate of 1 mm/min and an unloading rate of 2 mm/min. Five consecutive loading‐unloading cycles were performed within a strain range of 0%–60% to evaluate the fatigue resistance of the hydrogels.

Cylindrical hydrogel samples with a diameter of 20 mm and a height of 2 mm were prepared for rheological testing. Oscillatory frequency sweep measurements were performed on a rheometer within the range of 0.1–100 Hz at a fixed strain of 1%, while maintaining the temperature at 25°C.

### 5.8 Electrical Performance Testing

The electrochemical activity of the piezo‐mimetic ionic hydrogels (φ = 5 mm, h = 1 mm) was assessed using a potentiostat (Zennium, Zahner, Germany). Electrochemical impedance spectroscopy (EIS) was performed using a three‐electrode configuration on an electrochemical workstation to evaluate both ionic and electronic conductivity. The hydrogels were fixed on the working electrode in a standard three‐electrode setup. A platinum foil acted as the counter electrode, an Ag/AgCl electrode served as the reference electrode, and 1 mol/L H_2_SO_4_ solution (30 mL) was used as the electrolyte. Impedance measurements were conducted over a frequency range of 0.1–0.5 Hz for all hydrogel groups.

The electrical output performance was measured using a Keithley 6514 electrometer (Keithley Instruments, USA). Cylindrical hydrogels were subjected to different testing conditions by controlling displacement to achieve varying strain levels (e.g., 20%, 40%, 60%), as well as different loading conditions (5, 10, 15 N), and frequency with a custom‐built stepper motor. The output voltage, output current, and electrical resistance were recorded using a Keithley 6514 electrometer to evaluate the electrical performance of the hydrogels.

### 5.9 Finite Element Simulation of Piezoelectricity

A cylindrical geometric model of the piezo‐mimetic ionic hydrogel (φ = 15 mm, h = 5 mm) was created using CAD software. The geometry was discretized into hexahedral finite element meshes to ensure sufficient precision and resolution. The piezo‐mimetic ionic conductivity of the hydrogel was defined according to its measured ionic properties, while the elastic modulus and Poisson's ratio of PVA were assigned to describe the mechanical attributes. Electrode positions and geometries were specified, and boundary conditions were applied by imposing mechanical loading under different strain levels or electrical excitation through applied voltages to simulate the hydrogel response. Finite element simulations were performed using the ABAQUS solver to compute the system response, including electric field distribution and mechanical strain, for evaluation of the hydrogel performance.

### 5.10 Biocompatibility Assay in Vitro

Hydrogel samples sterilized by Co‐60 irradiation were placed into the tissue culture plates. BMSCs at passage 3 were harvested by enzymatic digestion to obtain a cell suspension, and 5000 cells per well were seeded onto the material surface. After allowing 4 h for cell attachment, culture medium was added, and the medium was subsequently refreshed every other day. Cell proliferation was evaluated on days 1, 4, and 7 using the Alamar Blue assay. On day 7, cell viability was further assessed by Live/Dead staining with calcein‐AM/PI.

### 5.11 Cell Migration Assay

Sterilized hydrogel samples were placed into the tissue culture plates. BMSCs at passage 3 were harvested by enzymatic digestion to obtain a cell suspension, and 10 000 cells per well were seeded onto the material surface. After allowing 4 h for cell attachment, culture medium was added, and the medium was refreshed every other day. On days 7 and 14, the samples were stained with calcein‐AM/PI and imaged by confocal laser scanning microscopy (CLSM, TCS SP5‐X, Leica, Germany) using z‐stack scanning to evaluate cell infiltration into the scaffolds. The migration distance of cells was quantified using IPP software.

### 5.12 Characterization of Chondrogenic Differentiation of BMSCs Under in Vitro Simulated Mechanical Stimulation

Passage 3 BMSCs were seeded at 10 000 cells per well onto sterilized cylindrical scaffolds (φ = 8 mm, h = 4 mm). After 4 h of attachment, culture medium was added. Following 3 days of culture, the proliferation medium was replaced with chondrogenic induction medium. Mechanical stimulation was applied daily to each scaffold using a custom‐built stepper motor system by controlling frequency and displacement, maintaining approximately 20% strain, for 5–10 min. From the first day of chondrogenic induction, culture was continued for 14 days. At day 14, the scaffolds were fixed with 4% paraformaldehyde and subjected to immunofluorescence staining for COL‐II and HIF‐1α. In parallel, qRT‐PCR analysis was performed to quantify the expression of chondrogenic‐related genes (COL‐II and SOX9), ion influx‐related genes (MAGT1, CAMK2D, and CALM1), and piezoelectric‐related genes (PIEZO), with primer sequences provided in Table S2.

### 5.13 Intracellular Mg^2+^ Staining

BMSCs (10 000 cells per well) were seeded on the surface of hydrogels, and after 7 days of cell infiltration into the scaffolds, mechanical stimulation was applied using a stepper motor for 10 min/day. Intracellular Mg^2+^ under different stimulation conditions was then analyzed using a Mg^2+^‐sensitive probe. Specifically, 4 µL of Mag‐Fluo‐4 AM stock solution (5 mm) and 5 µL of 20% Pluronic F‐127 were mixed with 1 mL of cell culture medium to prepare a staining solution. The working solution was further diluted and added to each group of cells, followed by incubation for 15–60 min. Finally, intracellular Mg^2+^ influx was visualized and imaged using CLSM at an excitation of 494 nm and an emission of 516 nm.

### 5.14 Piezo‐Mimetic Ionic Hydrogels Implantation in Rabbit Joint

New Zealand white rabbits used were obtained from the Experimental Animal Center of Peking University Health Science Center. All surgical procedures were performed in accordance with institutional guidelines for animal experimentation, following established standards of animal welfare and ethics. The protocol was approved by the Animal Ethics Committee of Peking University Health Science Center (Approval No. LA2022553).

For the fabrication of cartilage defects at different stress sites in vivo, rabbits weighing approximately 3.5 kg were selected, and defect models were created in both knees of each animal at the trochlea groove and the medial femoral condyle. The detailed experimental group assignments and modeling procedures are provided in Tables S3 and S4. The cartilage defects measured 3.5 mm in diameter and 2 mm in depth. At the trochlea site, two types of materials (PSG_c_ and PSG‐Mg_c_) were implanted, whereas PSG‐Mg_c_ samples were implanted at the femoral condyle site. The blank defect group and the Sham‐operated group were included as controls. After the PSG‐Mg_c_ group underwent implantation at the femoral condyle site, the in vivo piezoelectric response was recorded in real time. As the leg joint moved, the PSG‐Mg_c_ group generated electrical signals, which were then transmitted via a copper wire to a grounded electrometer.

At 8 weeks post‐implantation, the corresponding groups of experimental rabbits were euthanized and sampled. And then, the collected samples underwent MRI analysis to evidence the growth and knee effusion of new cartilage on a Siemens TIM Trio 3.0 T (T) MRI scanner (Siemens, Erlangen, Germany), using a small animal‐specific knee coil (Chenguang Medical Technologies, China) to improve the signal to noise and contrast to noise ratios. The protocol included three sequences for morphological observation and quantitative analysis. Total acquisition time was 20 min. Thereafter, the rabbits’ femoral joints were dissected and taken pictures, and then the repair of the defect was evaluated by the Mankin score (Table S5) and Wakitani score (Table S6) systems.

### 5.15 Phase Delay Analysis

At designated time points, knee joint samples were harvested from each group, and all soft tissues were carefully removed while maintaining consistent dissection procedures to avoid damaging the cartilage defect sites. The specimens were stored at −20°C until imaging. Prior to imaging, samples were thawed and equilibrated in a saline bath for 2 h. During imaging, a laser beam was directed perpendicularly onto the surface of the regenerated cartilage defect. All the samples were imaged by a custom‐built Jones‐matrix‐based PS‐OCT system. Histological imaging analysis was subsequently performed using a specific algorithm combined with MATLAB 2024b software to process and compare the samples.

### 5.16 Histology and Immunohistochemistry

The harvested knee samples were fixed in 10% neutral buffered formaldehyde for 72 h at RT, and decalcified in 10% formic acid solution with 10% neutral buffered formaldehyde at RT. The decalcified samples were trimmed, dehydrated in a graded ethanol series, and embedded in paraffin. Serial sections (8 µm thick) through the center of the repair site were cut and characterized by histological analysis and immunohistochemistry staining. The sections were subjected to H&E, Toluidine blue, Saf O/Fast green, and Alcian blue staining. Picrosirius Red was utilized to stain collagen, and directionality plugin from ImageJ software was used to determine the orientation distribution of the collagen fibers observed in the superficial and deeper zones of newly‐formed articular cartilage.

### 5.17 RNA Sequencing (RNA‐seq) Analysis of Neo‐Cartilage Tissue

To explore the underlying molecular mechanisms, identical full‐thickness cartilage defect models were established at both the trochlear groove and the medial femoral condyle. Defects were randomly assigned to three groups: the PSG_c_‐T (*n* = 4 defects) and PSG‐Mg_c_‐T (*n* = 4 defects) implanted at the trochlear groove, and the PSG‐Mg_c_‐C (*n* = 4 defects) implanted at the femoral condyle. At 8 weeks post‐implantation, rabbits were euthanized via CO_2_ asphyxiation, and the corresponding trochlear or femoral condylar tissues containing implanted hydrogels were harvested. Total RNA from the regenerated cartilage regions was isolated using TRIzol Reagent (Sigma, USA) following the manufacturer's protocol. After RNA purification, reverse transcription, and library preparation, whole‐transcriptome RNA‐seq was performed. Bioinformatic analyses, including Gene Ontology (GO) enrichment and Kyoto Encyclopedia of Genes and Genomes (KEGG) pathway analysis, were conducted on the Dr. Tom platform (https://biosys.bgi.com) provided by Beijing Genomics Institute (BGI, Shenzhen, China).

### 5.18 Statistical Analysis

All of the experimental data were measured in triplicate at least and were presented as mean ± standard deviation unless stated otherwise. Statistical analyses were performed using unpaired *t*‐tests for comparison of two groups of samples and ANOVA for multiple groups. Differences were considered significant at *p < 0.05, **p < 0.01, ***p < 0.001, ****p < 0.0001. All statistical analysis was performed using IBM SPSS Statistics 25.

## Author Contributions

**Chenyuan Gao**, **Wenli Dai**, and **Qing Cai**: conceptualization. **Chenyuan Gao**: data curation. **Chenyuan Gao** and **Yingjie Yu**: formal analysis. **Chenyuan Gao**, **Xinyu Wang**, **Wenli Dai**, and **Yingjie Yu**: visualization. **Chenyuan Gao**: writing – original draft. **Chenyuan Gao**, **Xinyu Wang**, **Zhifeng Wu**, **Xinya Zhang**, **Yue Wang**, **Xianbo Qiu**, **Cuiru Sun**, and **Wenli Dai**: methodology. **Chenyuan Gao**, **Xiao Geng**, **Ti Zhang**, **Hongwei Xia**, and **Jun Zhang**: investigation. **Hua Tian**, **Yingjie Yu**, and **Qing Cai**: supervision. **Chenyuan Gao** and **Qing Cai**: project administration. **Yingjie Yu** and **Qing Cai**: writing – review and editing. **Chenyuan Gao** and **Zhifeng Wu**: validation. **Chenyuan Gao**, **Hua Tian**, **Wenli Dai**, **Yingjie Yu**, and **Qing Cai**: funding acquisition. **Yingjie Yu** and **Qing Cai**: resources.

## Funding

This study was financially supported by the National Key Research and Development Program of China (2024YFC2418802), the National Natural Science Foundation of China (32371422, U22A20159, 82372450), Beijing Municipal Natural Science Foundation (L256033, L232092), and the Peking University Third Hospital Fund for Interdisciplinary Research (PT2416).

## Ethics Statement

The New Zealand white rabbits used for the cartilage orthotopic defect repair model in this study were obtained from the Experimental Animal Center of Peking University Health Science Center. All surgical procedures were conducted in accordance with institutional guidelines for animal experimentation and followed established standards of animal welfare and ethical practice. The protocol for the cartilage orthotopic defect repair model was approved by the Animal Ethics Committee of Peking University Health Science Center (Approval No. LA2022553).

## Consent

All participants and/or their legal representatives signed the written informed consent.

## Conflicts of Interest

The authors declare no conflicts of interest.

## Supporting information




**Supporting File 1**: advs75611‐sup‐0001‐SuppMat.docx.

**Supporting File 2**: advs75611‐sup‐0002‐MovieS1.mp4.

## Data Availability

The data that support the findings of this study are available from the corresponding author upon reasonable request.
