# Supplementary material for: A Piezo‐Mimetic Ionic Hydrogel Harnessing Joint Motion for Cartilage Repair
Source: Adv Sci (Weinh). 2026 May 10;13(43):e75611. doi: 10.1002/advs.75611 (PMC13336056; doi:10.1002/advs.75611)
Supplement: Supplementary file 1 — Supporting File 1: advs75611‐sup‐0001‐SuppMat.docx. [file ADVS-13-e75611-s001.docx]

**Supplementary Materials for**

**A Piezo-Mimetic Ionic Hydrogel Harnessing Joint Motion for Cartilage Repair**

Chenyuan Gao^1, 2^, Xinyu Wang^2^, Zhifeng Wu^3^, Xinya Zhang^4^, Xiao Geng^1^, Ti Zhang^1^, Yue Wang^2^, Hongwei Xia^2^, Jun Zhang^2^, Xianbo Qiu^3^, Cuiru Sun^4^, Yingjie Yu^2, *^, Wenli Dai^5, **^, Hua Tian^1, ***^, Qing Cai^2, ****^

^1^ Engineering Research Center of Bone and Joint Precision Medicine, Ministry of Education; Department of Orthopaedics, Peking University Third Hospital, Beijing 100191, China.

^2^ State Key Laboratory of Organic-Inorganic Composites, Beijing Laboratory of Biomedical Materials, Beijing University of Chemical Technology, Beijing 100029, China.

^3^ Institute of Microfluidic Chip Development in Biomedical Engineering, School of Information Science and Technology, Beijing University of Chemical Technology, Beijing 100029, China.

^4^ Department of Mechanics, Tianjin University, Tianjin 300072, China.

^5^ Institute of Sports Medicine, Beijing Key Laboratory of Sports Injuries, Peking University Third Hospital, Beijing 100191, China.

Corresponding to:

Prof. Qing Cai, E-mail: caiqing@mail.buct.edu.cn (Q. Cai)

Prof. Hua Tian, E-mail: tianhua@bjmu.edu.cn (H. Tian)

Dr. Wenli Dai, E-mail: wenlidai@pku.edu.cn (W. Dai)

Prof. Yingjie Yu, E-mail: yuyingjie@mail.buct.edu.cn (Y. Yu)

Table of contents

[**1.** **Supplementary Texts** 3](#_Toc219983871)

[Text S1. Synthetic characterization of Acy-Gel 3](#_Toc219983872)

[Text S2. Characterization of mechanical properties of piezo-mimetic ionic hydrogels 3](#_Toc219983873)

[Text S3. Characterization of cation binding and ion release behavior of piezo-mimetic ionic hydrogels 5](#_Toc219983874)

[Text S4. Electrochemical properties of Piezo-mimetic Ionic Hydrogels 6](#_Toc219983875)

[Text S5. Cytotoxicity evaluation of Piezo-mimetic Ionic Hydrogels 7](#_Toc219983876)

[**2.** **Supplementary Figures** 9](#_Toc219983877)

[**3.** **Supplementary Tables** 27](#_Toc219983878)

[**4.** **Supplementary Reference** 31](#_Toc219983879)

1. **Supplementary Texts**

**Text S1. Synthetic characterization of Acy-Gel**

As shown in the FT-IR spectra (**Figure. S1**), characteristic absorption peaks at 1650 cm^-1^ and 1540 cm^-1^ correspond to the amide I and amide II bands, respectively. Comparison of spectra from gelatin (Gel) and acylated gelatin (Acy-Gel) revealed the appearance of a new medium-intensity peak at 1400 cm^-1^ in Acy-Gel, corresponding to the symmetric stretching vibration of carboxyl groups. This indicates that succinic anhydride modification successfully introduced additional carboxyl moieties. Furthermore, both Gel and Acy-Gel exhibited the characteristic protein absorption bands of amide I and II with nearly identical peak positions, confirming that the acylation process did not alter the fundamental peptide backbone structure of gelatin.

**Text S2. Characterization of mechanical properties of piezo-mimetic ionic hydrogels**

A kind of interpenetrating network was constructed by incorporating Acy-Gel into a PVA and SA mixed matrix. Subsequent ionic crosslinking with divalent cations (Ca^2+^ or Mg^2+^) produced ionically conductive composite hydrogels, while PBS-soaked samples served as controls. Due to multiple intermolecular interactions-including extensive hydrogen bonding and electrostatic attractions-the resulted ion-conductive hydrogels exhibited good mechanical performance. The tensile and rheological properties of the three hydrogels under different solution conditions are summarized in **Figure. S3**. As shown in **Figure. S3A**, without ionic crosslinking (PBS-soaked samples), the incorporation of Acy-Gel markedly enhanced the elongation at break of the PG hydrogel (≈110%), compared with only ≈60% for the PS hydrogel. The PSG hydrogel, containing both SA and Acy-Gel, showed an intermediate elongation of ≈80%, confirming that Acy-Gel improved the toughness of the polymer network. PVA, a linear polymer with good film-forming and water-soluble properties, and SA, a natural polysaccharide with excellent biocompatibility and gelation capacity, interact through intensive hydrogen bonding to form a robust three-dimensional PS network with high mechanical strength. Acy-Gel functions as a flexible secondary network that interpenetrates this rigid PS framework, creating a dual-network PSG hydrogel. In this design, the SA content was reduced by half compared with the PS hydrogel, while Acy-Gel was incorporated to further optimize the hydrogel’s performance. The resulting architecture enhances toughness and elasticity, improves resistance to deformation and impact, and effectively integrates the high strength of the PS network with the high ductility of the Acy-Gel network, thereby achieving an optimized mechanical profile suitable for cartilage repair applications.

Following ionic crosslinking (**Figure. S3B-C**), all groups showed increased tensile strength. Specifically, the Ca^2+^-crosslinked PS hydrogel exhibited a fracture stress of 105 kPa but a reduced elongation (< 40%). In contrast, the Mg^2+^-crosslinked PG hydrogel displayed a fracture stress of 16 kPa and an elongation of ≈80%, indicating stronger Mg^2+^ crosslinking and a more pronounced enhancement in toughness. The PSG-Mg hydrogel exhibited mechanical properties between those of the PS and PG groups; compared with its Ca^2+^-crosslinked counterpart, the presence of gelatin improved ductility, suggesting superior suitability for cartilage applications. Rheological tests (**Figure S3D-F**) revealed that the storage modulus (G’) and loss modulus (G’’) of PS, PG, and PSG hydrogels remained stable. All groups maintained mechanical stability after ionic crosslinking, and G’ increased in each case. The modulus consistently followed the trend PS > PSG > PG under all crosslinking conditions, further corroborating the tensile testing results.

Articular cartilage serves as the primary load-bearing tissue in joints, providing essential functions such as load transmission, shock absorption, and wear resistance. Elastic recovery is a critical factor that enables cartilage to withstand repeated mechanical loading. As shown in **Figure S4** and **Movie S1**, the cyclic compression behavior of the three hydrogel groups was evaluated within a strain range of 0-60% after different ionic crosslinking treatments. Analysis of the stress-strain curves revealed that the PVA-based composite hydrogels exhibited excellent resilience and stable mechanical performance. Further comparison between Ca^2+^- and Mg^2+^-crosslinked samples indicated that although the PS group showed a significant increase in compressive stress after ionic crosslinking, its recoverability was inferior to that of the PG and PSG groups. This reduced elasticity was attributed to the highly rigid polymer network of PS, which became even stiffer after ionic crosslinking, thereby limiting its ability to return to the original shape after the first compression cycle. In contrast, the PG hydrogels demonstrated favorable compressive resilience, and their mechanical strength was enhanced more effectively by Mg^2+^ crosslinking than by Ca^2+^. Benefiting from the synergistic presence of SA and Acy-Gel, the PSG hydrogels compensated for the mechanical shortcomings of both PS and PG, achieving superior mechanical strength and compressive recovery. Benefiting from the rigid ion-crosslinked segments provided by SA and the flexible ion-responsive domains introduced by Acy-Gel, the PSG hydrogels exhibit superior mechanical strength and tensile toughness after crosslinking with either Ca^2+^ or Mg^2+^. Also, PSG-Mg exhibited greater mechanical stability, better resilience, and enhanced fatigue resistance. As shown in **Figure S4B**, during the fifth compression cycle to 60% strain, the PSG-Mg hydrogel rapidly released its stored elastic energy and returned promptly to its original configuration once the load was removed.

**Text S3. Characterization of cation binding and ion release behavior of piezo-mimetic ionic hydrogels**

To evaluate the respective ionic binding affinities of SA and Acy-Gel toward Ca^2+^ and Mg^2+^, ion adsorption experiments were performed using corresponding salt solutions, as shown in **Figure S5**. The PS, PG, and PSG hydrogel samples were individually immersed in CaCl_2_ and MgCl_2_ solutions of known concentrations (0.5 M). After reaching equilibrium crosslinking, the hydrogels were removed, and the residual ion concentrations in the solutions were measured. Quantitative analysis revealed that the PG group exhibited a stronger affinity for Mg^2+^, whereas the PS group showed a higher binding capacity for Ca^2+^. The PSG hydrogels, containing both SA and Acy-Gel components, displayed intermediate binding abilities toward Ca^2+^ and Mg^2+^ compared with the individual PS and PG systems. These findings confirm that the Acy-Gel component in the PG polymer network enhances complexation with the smaller ionic radius of Mg^2+^ (0.57 Å), while the SA-based PS network preferentially coordinates with the larger ionic radius of Ca^2+^ (1.00 Å) [1]. This behavior can be explained by the classical “egg-box” model of SA, in which the G-blocks (guluronic acid regions) form well-defined chain cavities that preferentially accommodate larger divalent cations such as Ca^2+^, thereby generating more stable ionic crosslinks [2, 3]. In contrast, Mg^2+^ possesses a smaller ionic radius and a significantly stronger hydration shell, making it energetically unfavorable for the ion to dehydrate and intercalate into the egg-box cavities [4]. As a result, Mg^2+^ typically exhibits much lower crosslinking efficiency in conventional alginate-Ca^2+^ systems. Conversely, acylated gelatin and other carboxyl-rich flexible polymers provide a more open and deformable network architecture, where the smaller Mg^2+^ ion has greater coordination freedom and can more readily access and bind to exposed carboxyl groups. This structural flexibility enables more effective Mg^2+^-carboxyl coordination, resulting in more favorable ionic crosslinking within the Acy-Gel-containing hydrogel network. Besides, the smaller size and higher charge density of Mg^2+^ facilitate stronger electrostatic interactions and chelation.

On the other hand, the cumulative release profiles of Ca^2+^ and Mg^2+^ over 14 days were further analyzed (**Figure S6**). The PS hydrogels exhibited the lowest cumulative Ca^2+^ concentration, indicating a stronger binding affinity toward Ca^2+^ with SA (**Figure S6A**). In contrast, the PG group showed weaker Ca^2+^ binding, leading to rapid ion release and the highest Ca^2+^ concentration in the aqueous phase. The PSG hydrogels demonstrated an intermediate Ca^2+^ release behavior between PS and PG, reflecting a balanced ion-binding capacity. Regarding Mg^2+^ in **Figure S6B**, the PS group displayed the highest cumulative release, consistent with its weaker affinity for Mg^2+^. Both PG and PSG exhibited comparable Mg^2+^ release profiles, suggesting that in the PSG system, Mg^2+^ coordination primarily occurred through the carboxyl groups of Acy-Gel.

**Text S4.** **Electrochemical properties of Piezo-mimetic Ionic Hydrogels**

In addition to enhancing the mechanical properties of the hydrogel network, multifunctional Ca^2+^ and Mg^2+^ also improved the ionic conductivity of the hydrogels. The Nyquist plots of the PS, PG, and PSG hydrogels equilibrated in CaCl_2_ and MgCl_2_ solutions are presented in **Figure S7A**. The corresponding ionic conductivities calculated from the results of the Nyquist plots are shown in **Figure S7B**, with the hydrogels soaked in PBS serving as the control. As observed in the **Figure S7A-B**, the Mg^2+^-equilibrated hydrogels exhibited the lowest impedance values and the highest ionic conductivity, indicating superior electrical conductivity. This enhancement arises because Mg^2+^, which has a smaller ionic radius and a more strongly bound hydration shell than Ca^2+^. And its incorporation into polymer chains requires higher dehydration energy. As a result, Mg^2+^ forms predominantly monodentate coordination with carboxyl groups rather than strong multidentate crosslinks, leading to a more relaxed and loosely crosslinked polymer network. Consequently, the Mg^2+^-crosslinked hydrogels retain a larger population of mobile free ions, which generates more pronounced ionic conductivity signals. Note that the PBS-equilibrated hydrogels possessed a relatively lower conductivity (**Figure S7B**), attributable to the limited ionization of carboxyl groups and the correspondingly low concentration of free ions within the hydrogel network.

Subsequently, the output current of the PS, PG, and PSG hydrogels equilibrated in different ionic solutions was measured (**Figure S8**). For the samples soaked in PBS (**Figure S8A**), all three groups exhibited minor current fluctuations under mechanical stimulation, with no significant differences among them. In contrast, after Ca^2+^ or Mg^2+^ equilibrated (**Figure S8B-C**), the hydrogels showed distinct current responses, with the PG group producing the most pronounced signals, particularly in the PG-Mg group (PG-Mg > PG-Ca). This behavior can be attributed to the Acy-Gel in PG, which provides abundant carboxyl groups that enhance Mg^2+^ coordination. However, because Mg^2+^ primarily forms monodentate coordination with the polymer chains, the resulting network is more loosely cross-linked. This relaxed structure allows a higher concentration of mobile ions to remain within the network, facilitating enhanced ion migration during deformation and thereby generating stronger ionic current outputs. The PSG-Mg hydrogel also generated higher output currents than PSG-Ca. This difference suggests that in PSG-Ca, the network is dominated by strong Ca^2+^-SA ionic crosslinks, which restrict ion mobility and yield weaker current signals. Conversely, in the PSG-Mg hydrogel, both SA and Acy-Gel coordinate Mg^2+^ to form a more loosely organized, high-mobility crosslinking network. This relaxed ionic structure retains more mobile, thereby strengthening ion migration under deformation and enhancing the overall piezo-ionic effect.

**Text S5. Cytotoxicity evaluation of Piezo-mimetic Ionic Hydrogels**

Based on our previous studies [5-7], functional Mg^2+^ plays a crucial role in promoting stem cells’ chondrogenic differentiation and attenuating osteoarthritic progression. These results therefore provide further support for the potential of the ionically conductive hydrogel system developed in this study for cartilage regeneration and repair. And, the cell compatibility assays further supported these findings (**Figure S9**). Although Ca^2+^ crosslinking did not exhibit cytotoxicity, cell proliferation on these hydrogels was markedly slower compared with the PBS- and Mg^2+^-treated groups. This effect became more evident with prolonged culture, likely due to the sustained release of Ca^2+^. Excessive Ca^2+^ levels are known to suppress cell proliferation. Wang et al. reported that when the local Ca^2+^ concentration exceeds approximately 100 ppm, a marked inhibitory effect on cell growth occurs [8]. Based on our ion-release measurements (**Figure S6**), concentration estimates indicate that even the PS-Ca^2+^ group, which exhibited the lowest Ca^2+^ release, reached an average concentration close to 100 ppm within 5 days of culture. In contrast, Mg^2+^ exhibited a higher biological tolerance threshold, maintaining normal viability and differentiation of both stem cells and chondrocytes even at elevated concentrations. Taken together, the PSG-Mg hydrogel demonstrated optimal overall performance. It combines the mechanical robustness of the PVA matrix, the rigidity of SA segments, and the toughness of Acy-Gel chains, while also exhibiting enhanced ionic conductivity, evident current output under deformation, and favorable cytocompatibility. Therefore, the PSG-Mg hydrogel was selected for subsequent in-depth investigation and functional evaluation.

1. **Supplementary Figures**


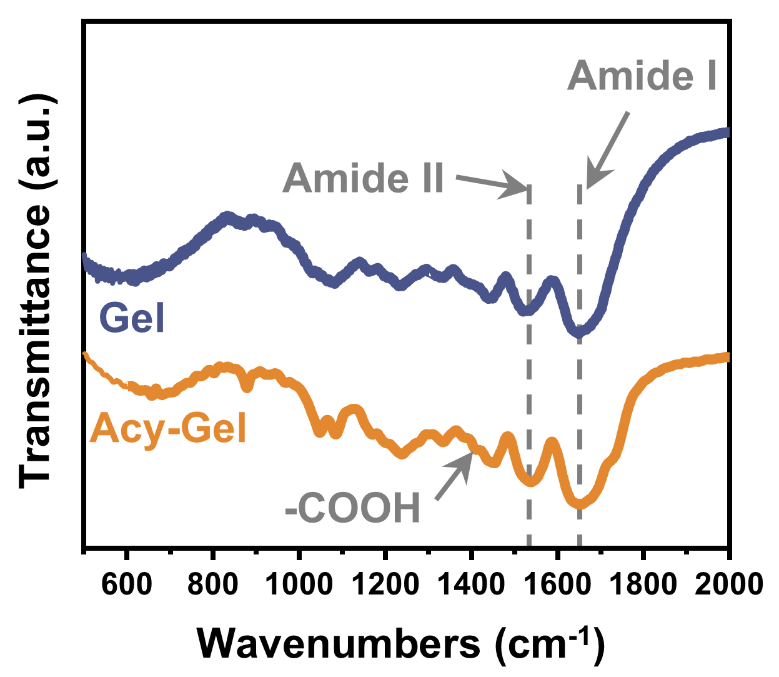


**Figure S1.** FT-IR spectra of Gel and Acy-Gel.


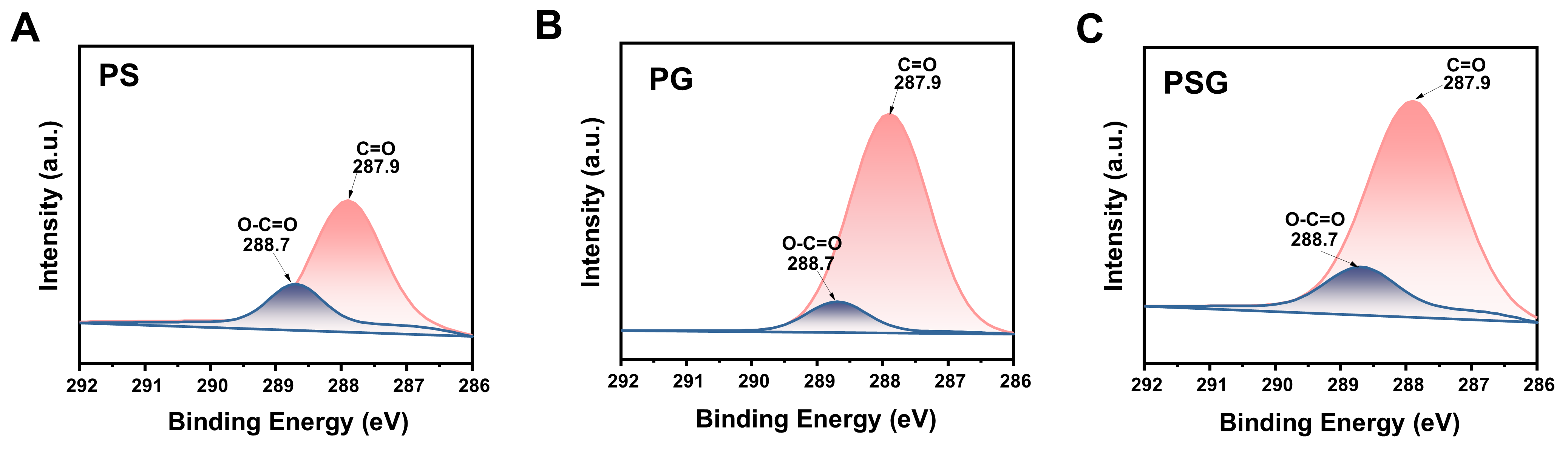


**Figure S2.** XPS C1s spectra of PS, PG, and PSG hydrogel formulations.


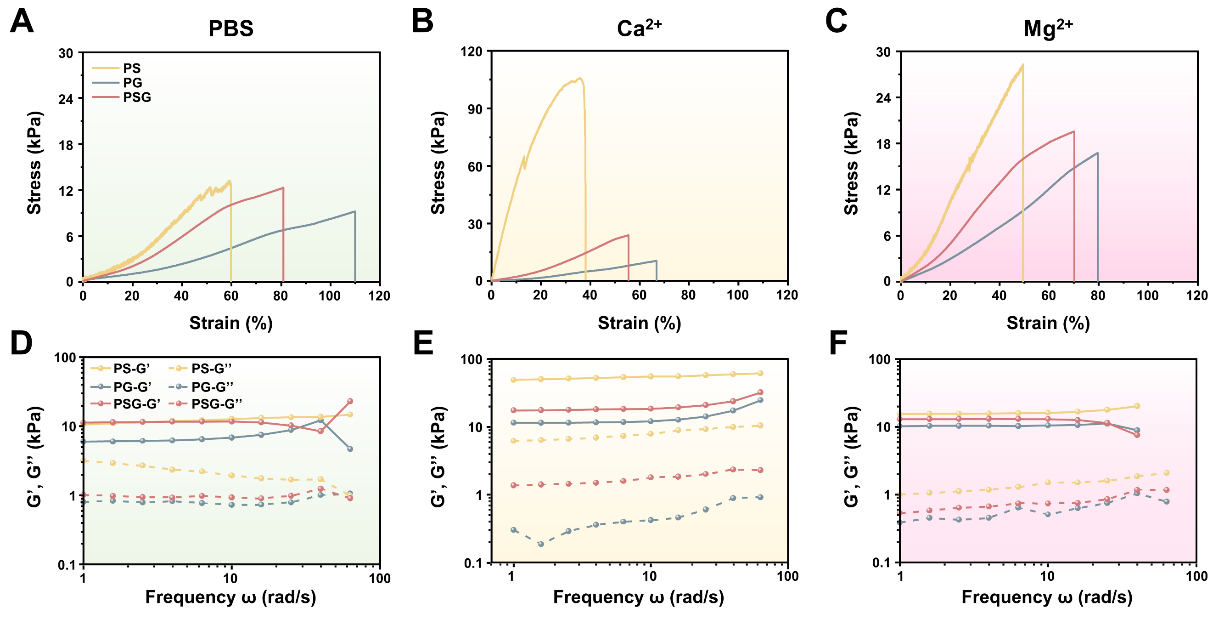


**Figure S3.** Tensile mechanics (**A-C**) and rheology (**D-F**) of PS, PG and PSG hydrogels after being equilibrated in different ionic solutions.


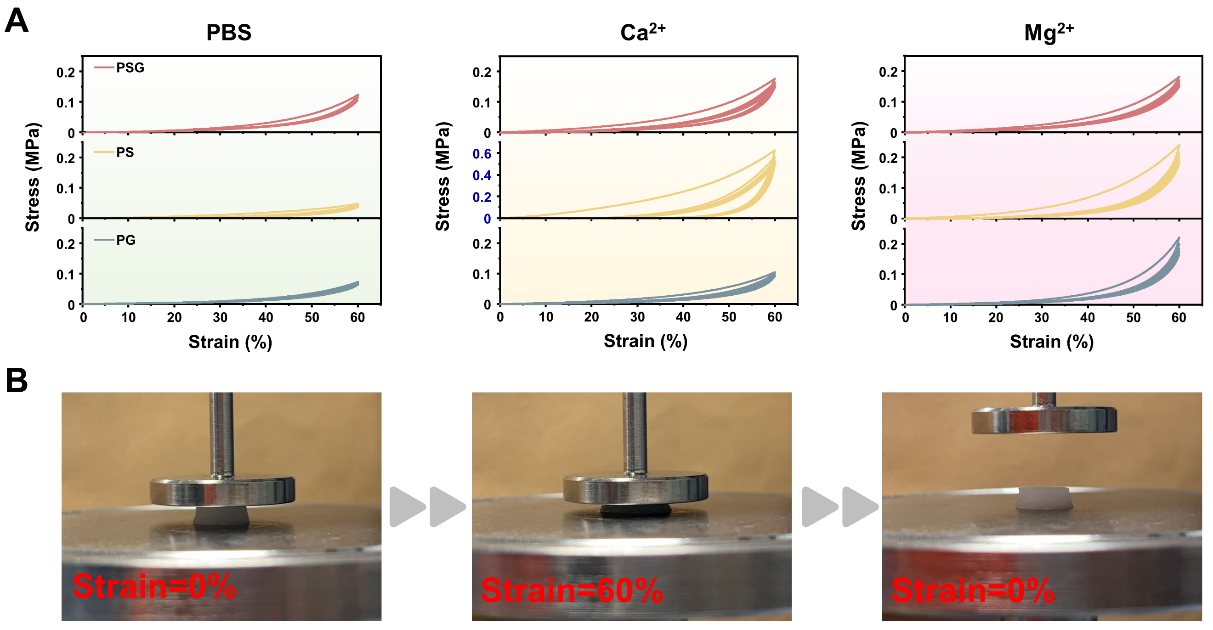


**Figure S4**. Cyclic compression tests of PS, PG, and PSG hydrogels equilibrated in different ionic solutions. (**A**) Stress-strain curves. (**B**) Macroscopic photographic record of PSG-Mg during the 5th cycle.


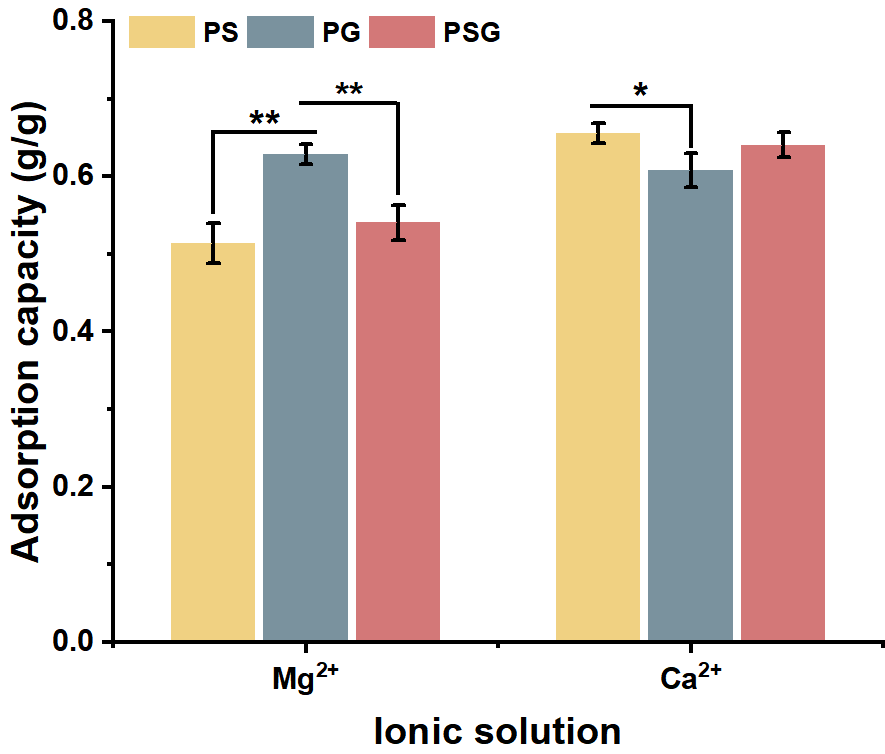


**Figure S5.** Characterization of uptake of Mg^2+^ and Ca^2+^ by PS, PG and PSG hydrogels. Results are presented as mean ± SD (n ≥ 3). ^*^p < 0.05, ^**^p < 0.01.


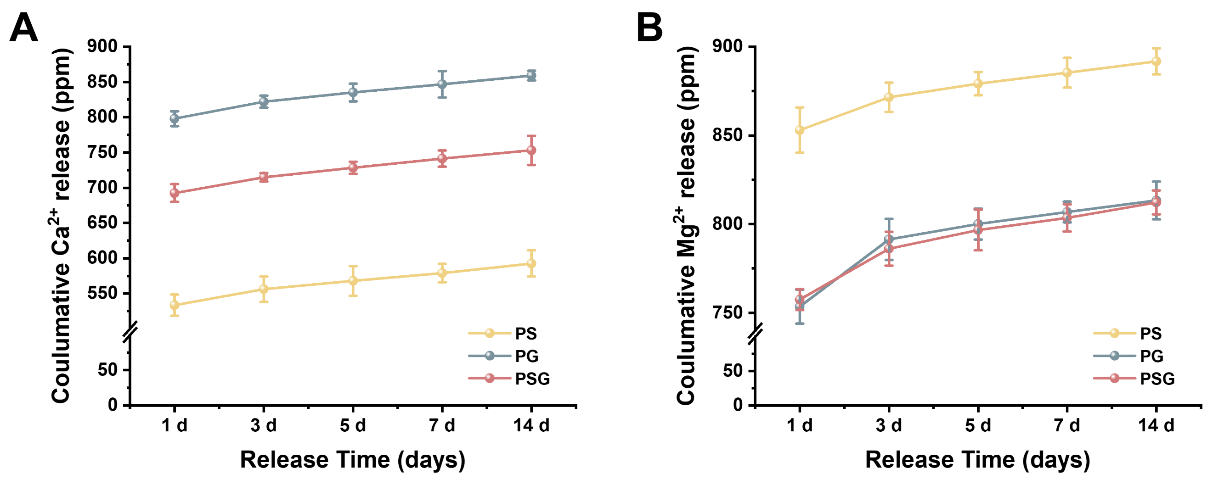


**Figure S6.** Characterization of release capacity of Mg^2+^ and Ca^2+^ by PS, PG and PSG hydrogels. (**A**) Cumulative release concentration of Ca^2+^. (**B**) Cumulative release concentration of Mg^2+^.


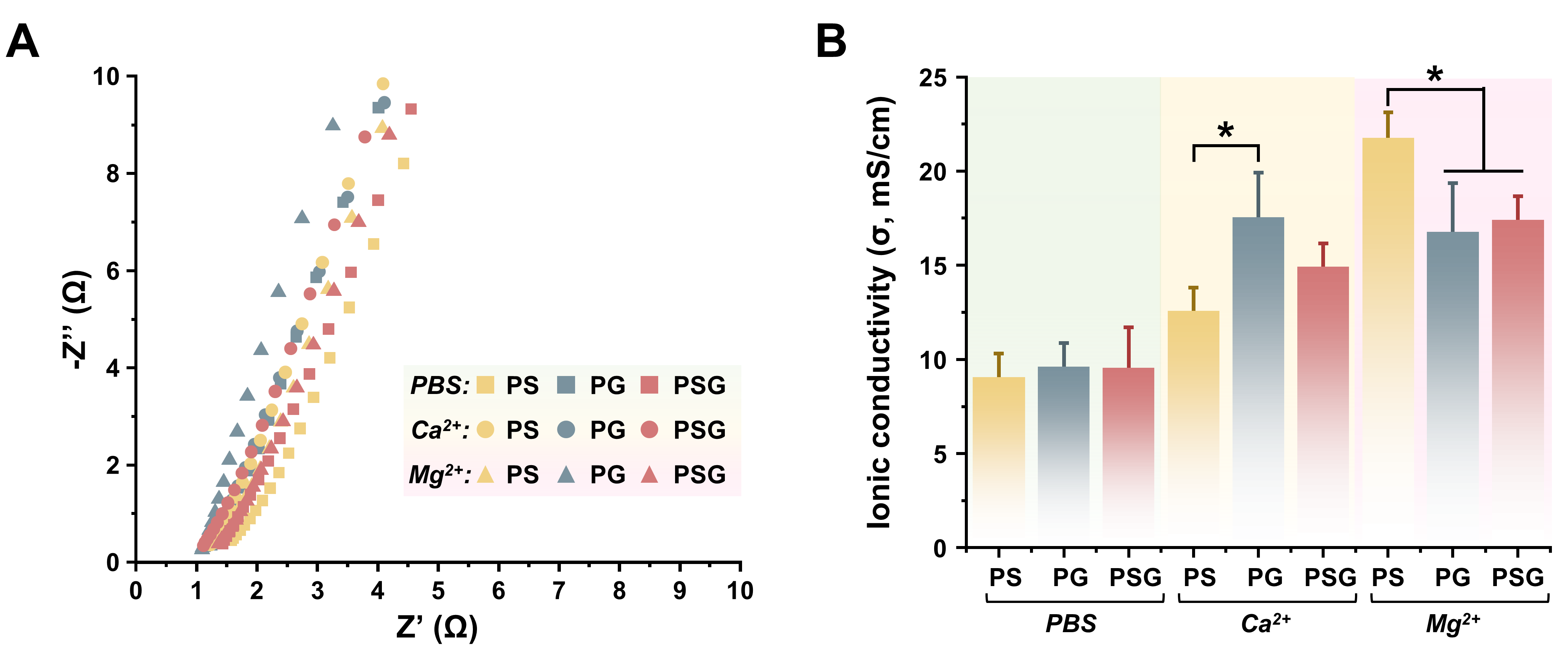


**Figure S7.** Nyquist plots of the PS, PG and PSG hydrogels equilibrated in different ionic solutions (**A**), and the corresponding ionic conductivities calculated from the impedance spectra (**B**). Results are presented as mean ± SD (n ≥ 3). ^*^p < 0.05.


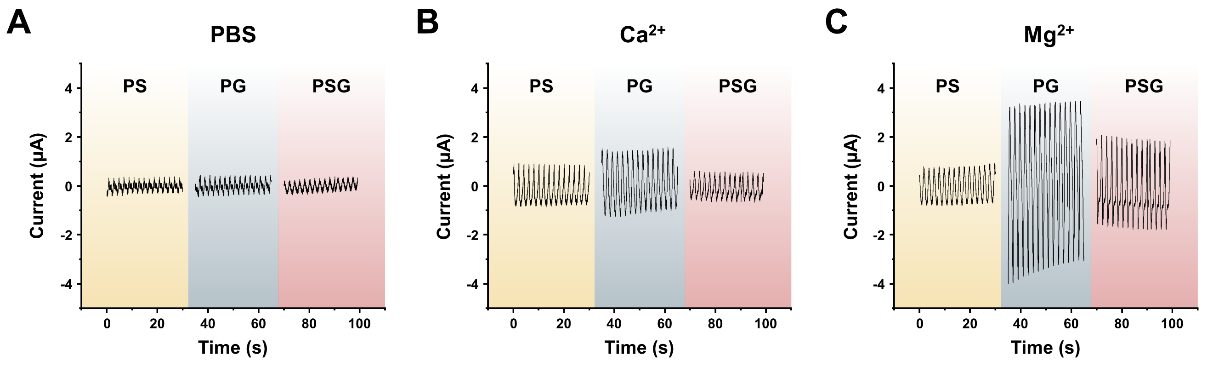


**Figure S8**. Output currents generated by deformation under pressure of PS, PG and PSG with different ion’s cross-linking solutions: (**A**) PBS; (**B**) Ca^2+^; (**C**)Mg^2+^.


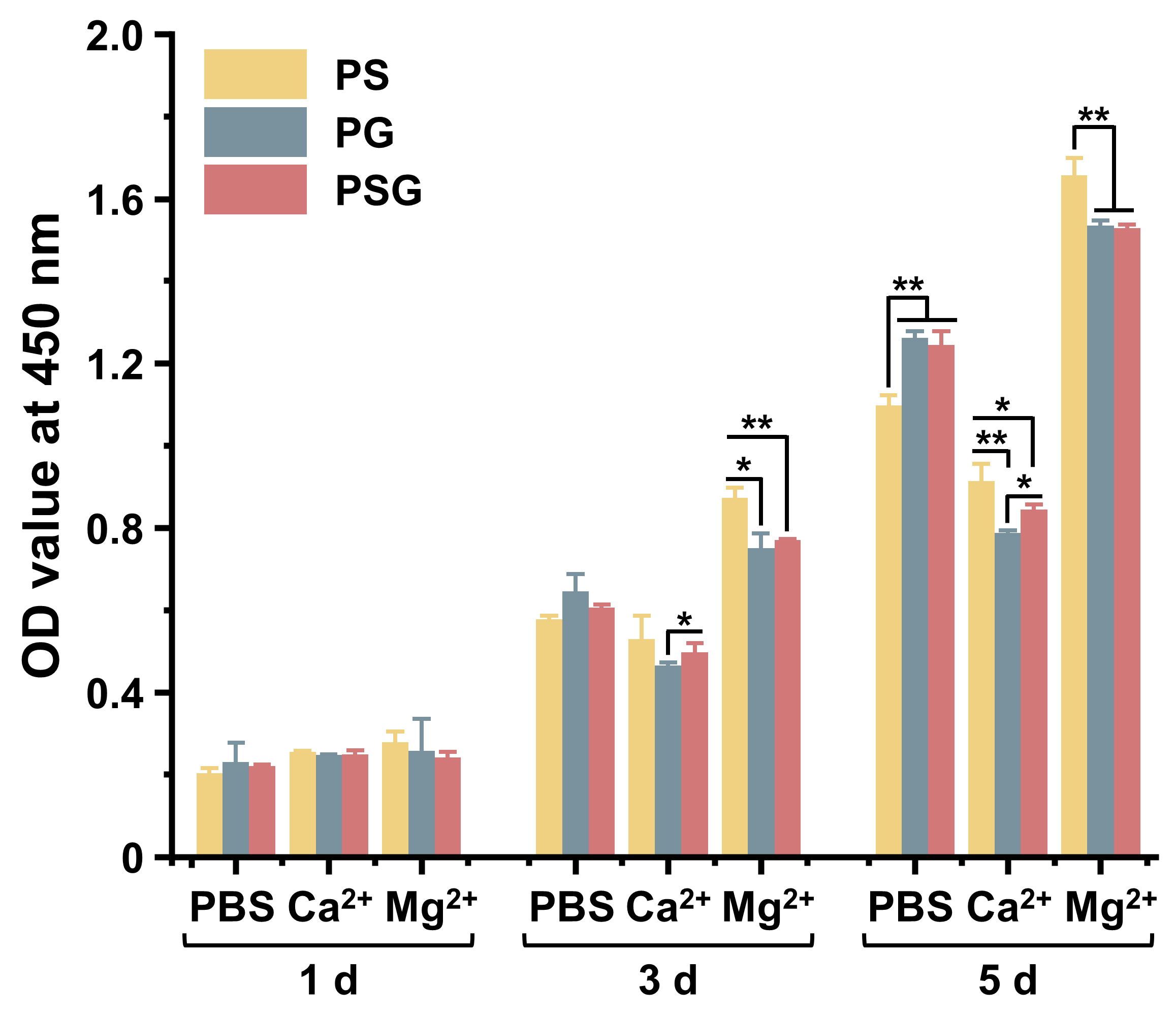


**Figure S9**. Cytotoxicity testing of PS, PG and PSG hydrogels equilibrated in different ionic solutions (PBS, Ca^2+^, and Mg^2+^) using BMSCs. Results are presented as mean ± SD (n ≥ 3). ^*^p < 0.05, ^**^p < 0.01.


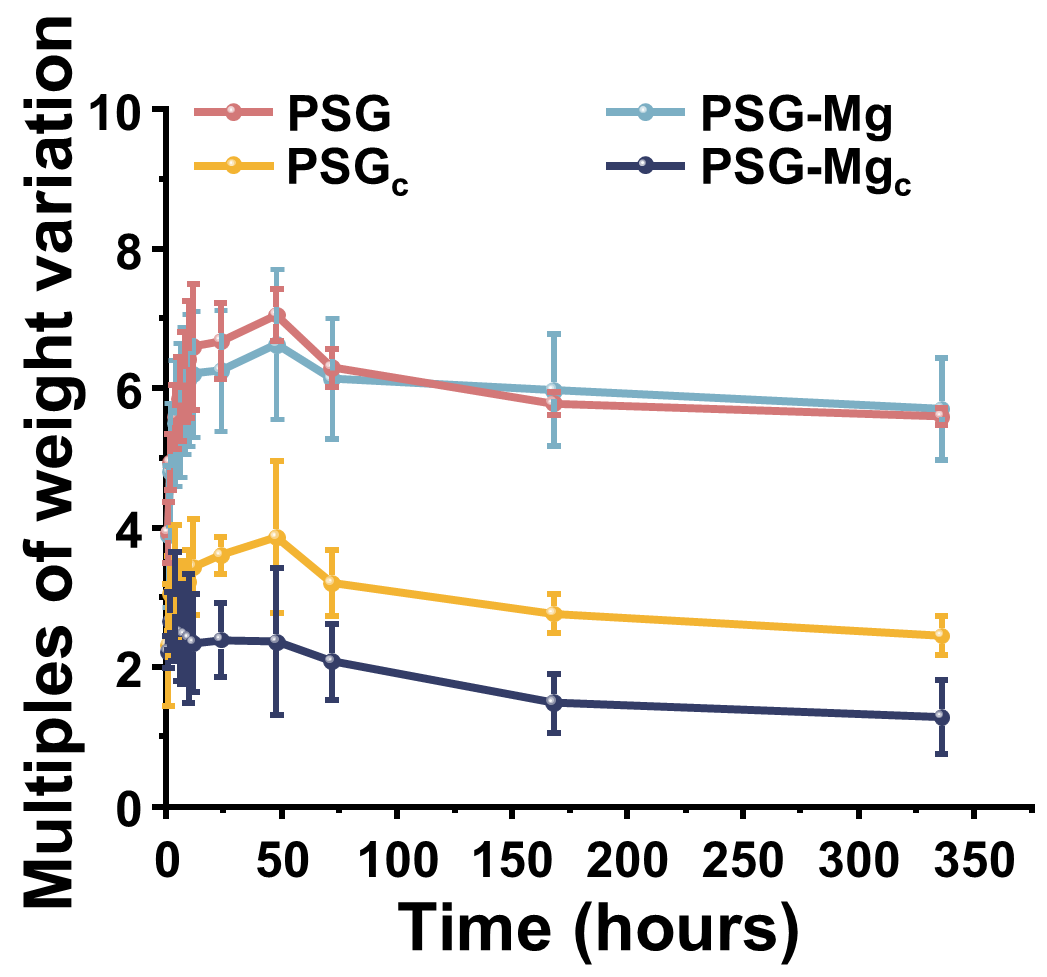


**Figure S10**. Evaluation of EDC/NHS treatment on the degradation of PSG and PSG-Mg hydrogels.


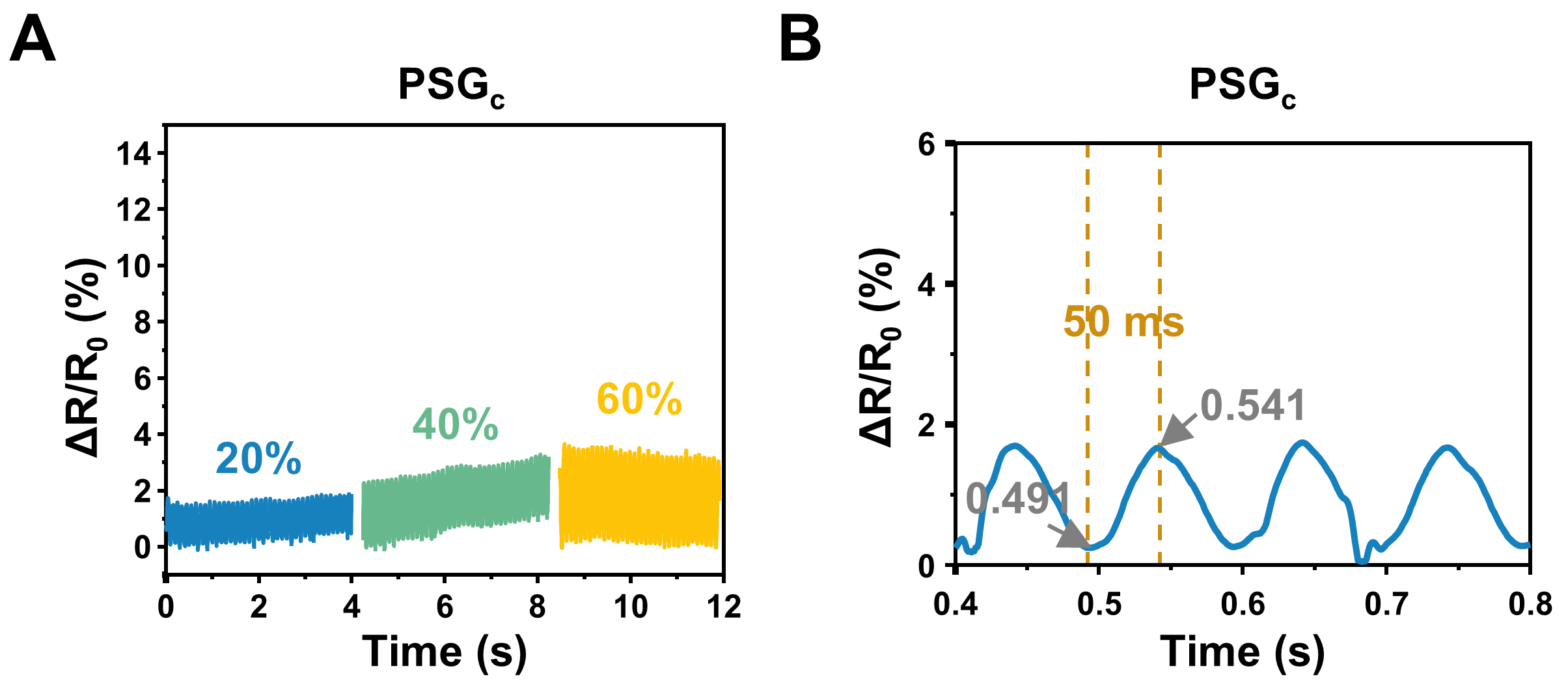


**Figure S11**. Cyclic performance of the PSG_c_ hydrogel’s sensitivity at the strain of 20%, 40% and 60% (**A**), and response patterns of PSG_c_ hydrogel under 20% compressive deformation (**B**).


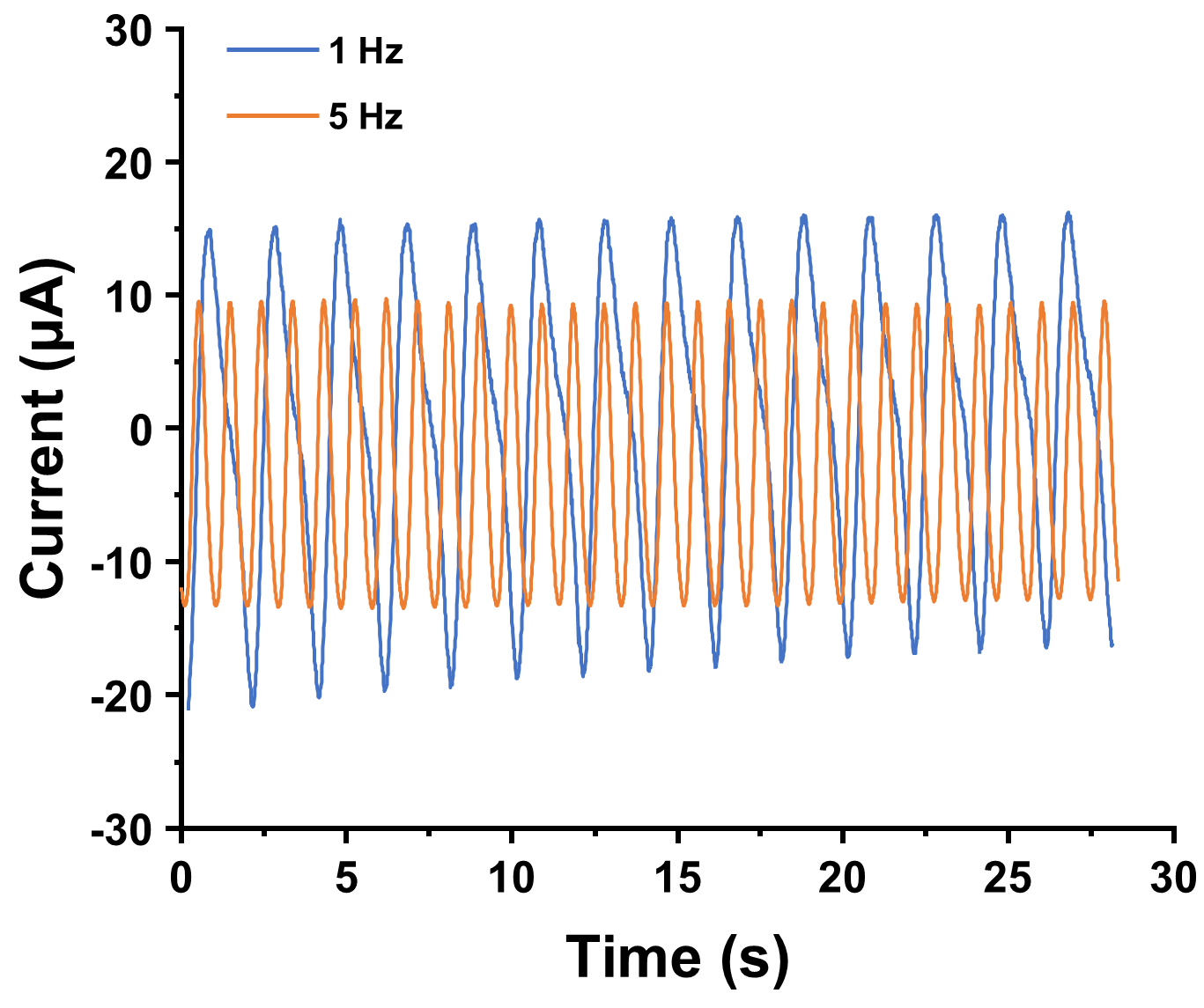


**Figure S12**. Piezo-mimetic current output of PSG-Mg_c_ hydrogel under cyclic mechanical loading at different frequencies (1 Hz and 5 Hz).


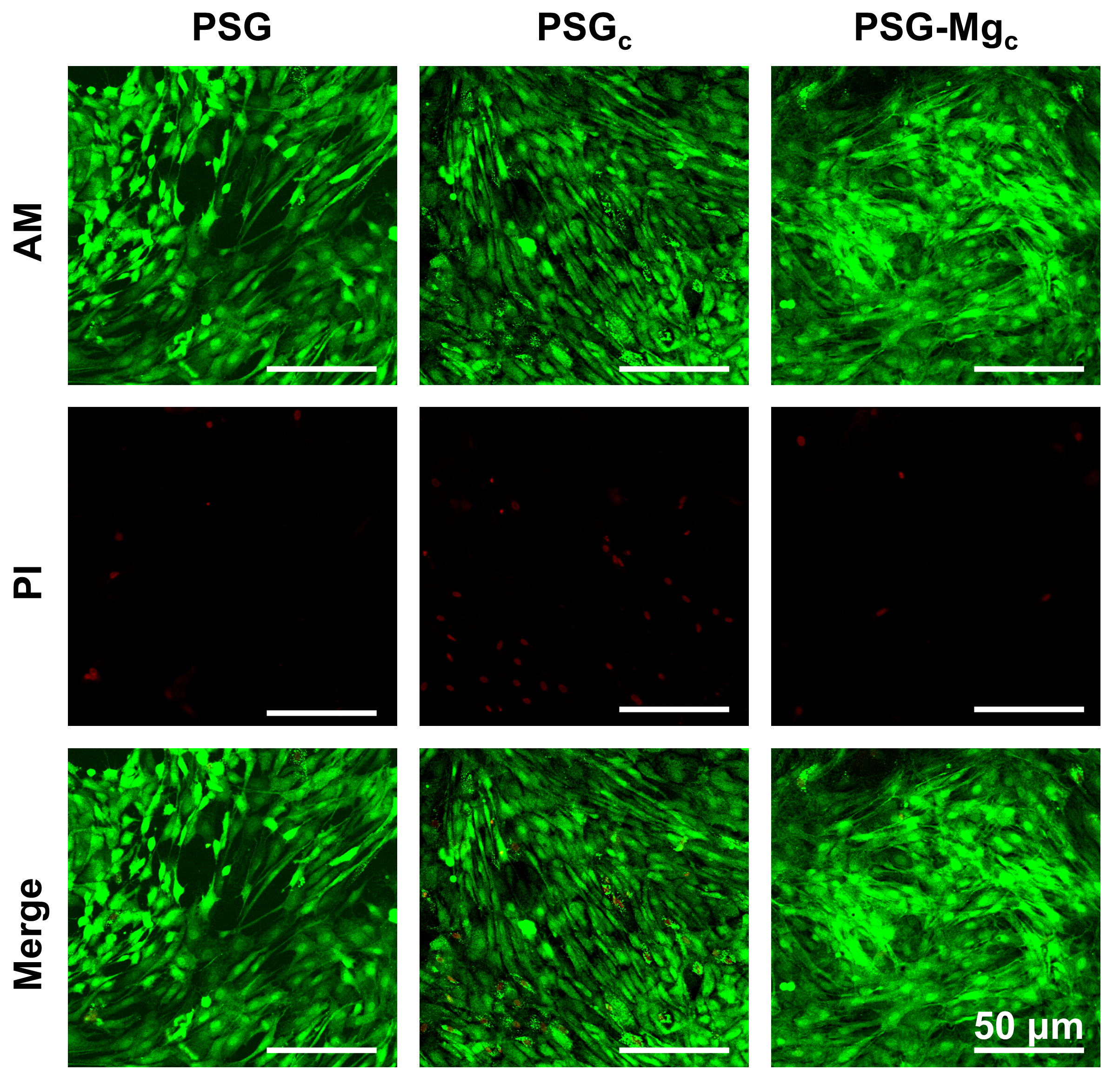


**Figure S13**. Live-dead staining of MSCs cultured on PSG, PSG_c_ and PSG-Mg_c_ hydrogels for 7 days.


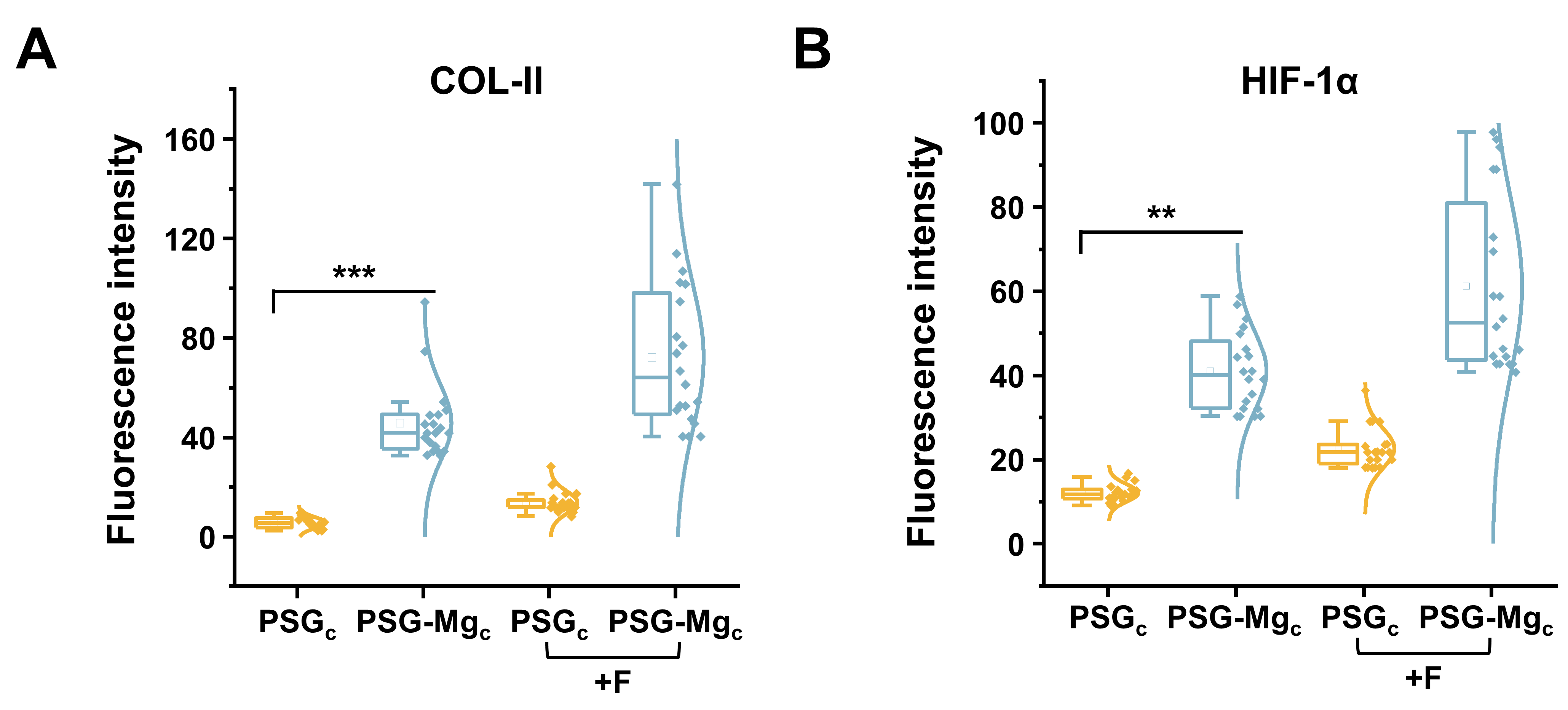


**Figure S14**. Semiquantitative analysis on fluorescence intensity for COL-II (**A**) and HIF-1α (**B**). Results are presented as mean ± SD (n ≥ 3). ^**^p < 0.01, ^***^p < 0.001.


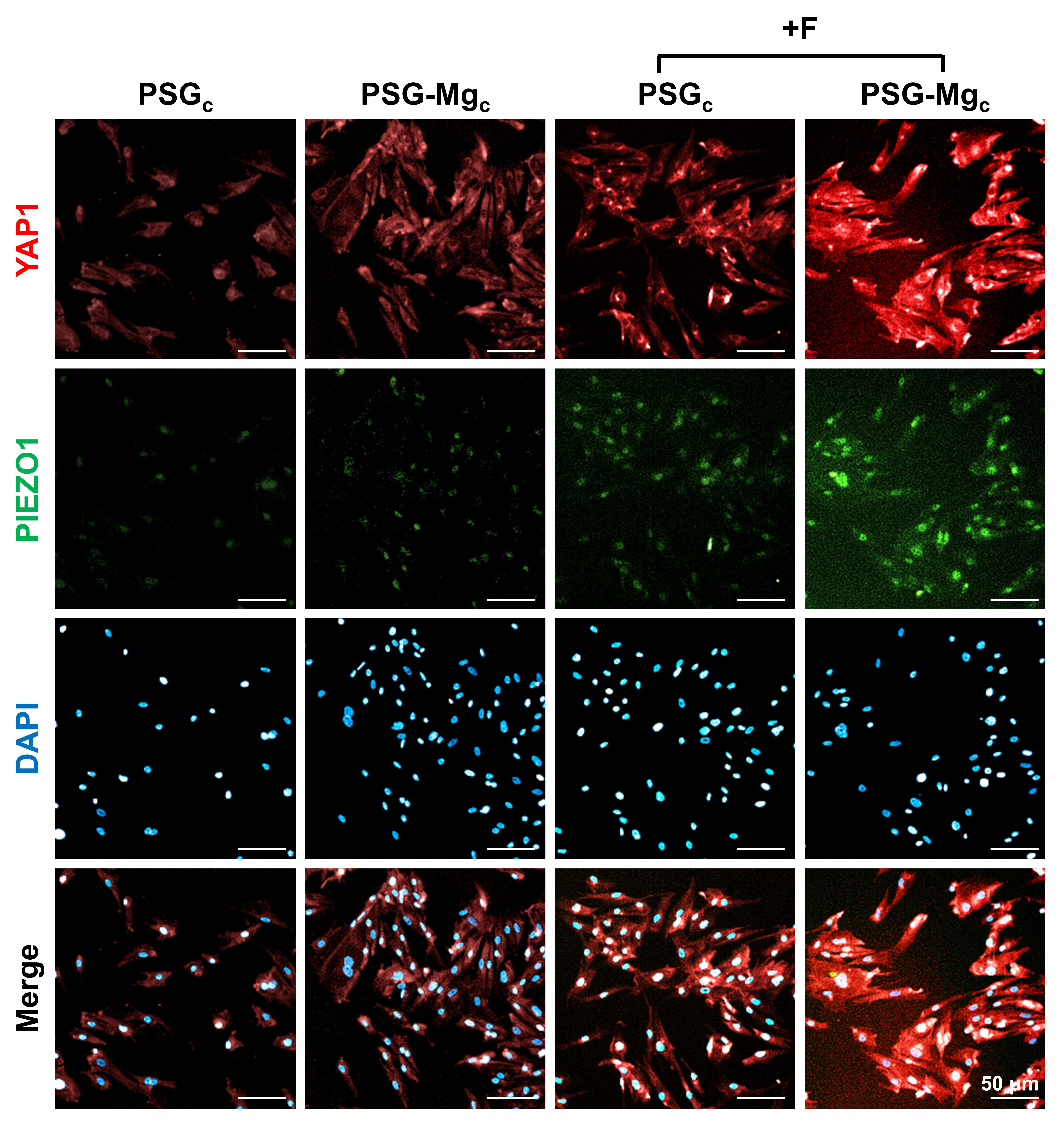


**Figure S15.** Co-immunofluorescence analysis of YAP and PIEZO1 in BMSCs cultured on PSG_c_ and PSG-Mg_c_ hydrogels, with or without the mechanical stimulation.


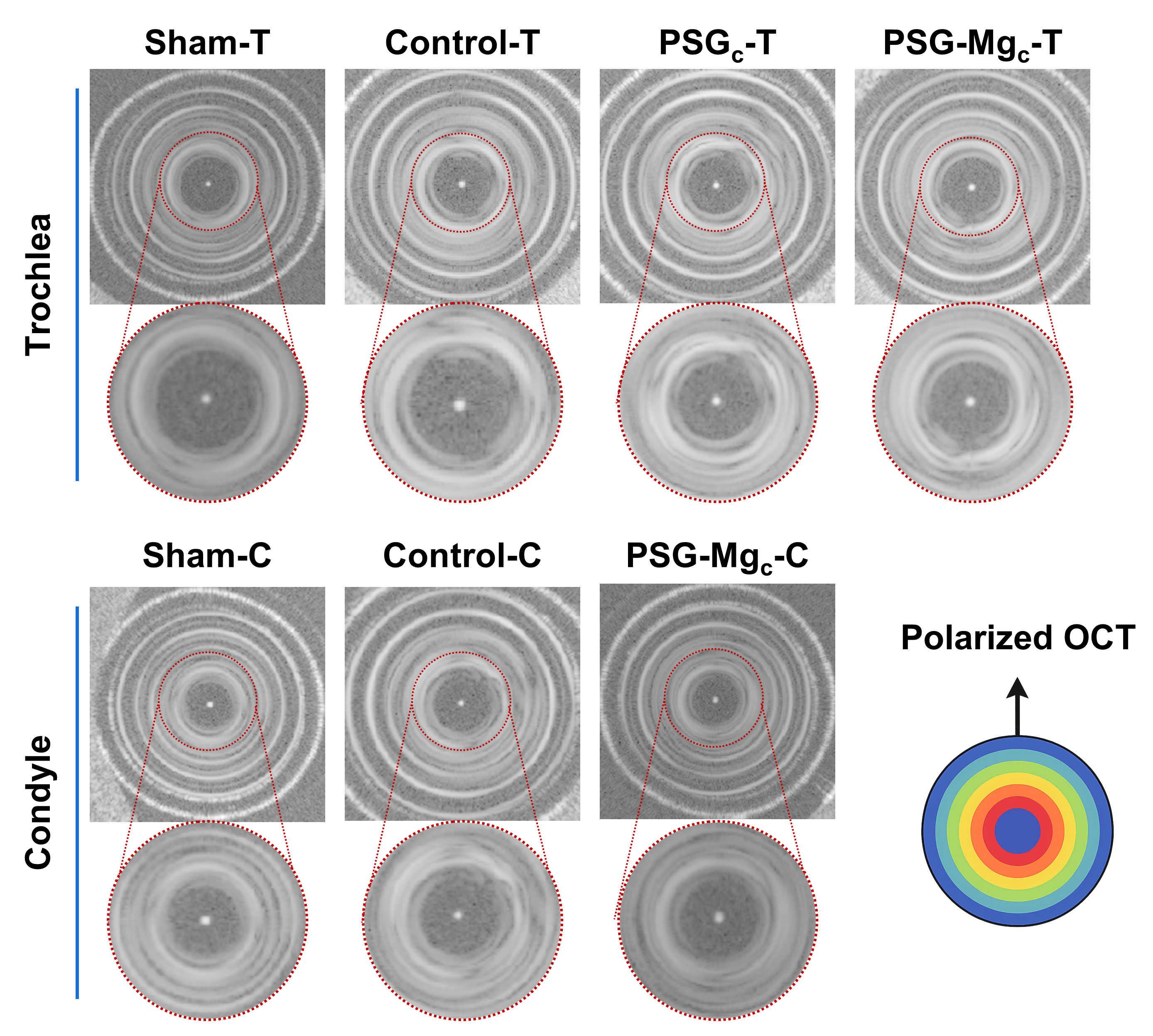


**Figure S16.** Polarization-sensitive OCT retardation images of cartilage repair sites.


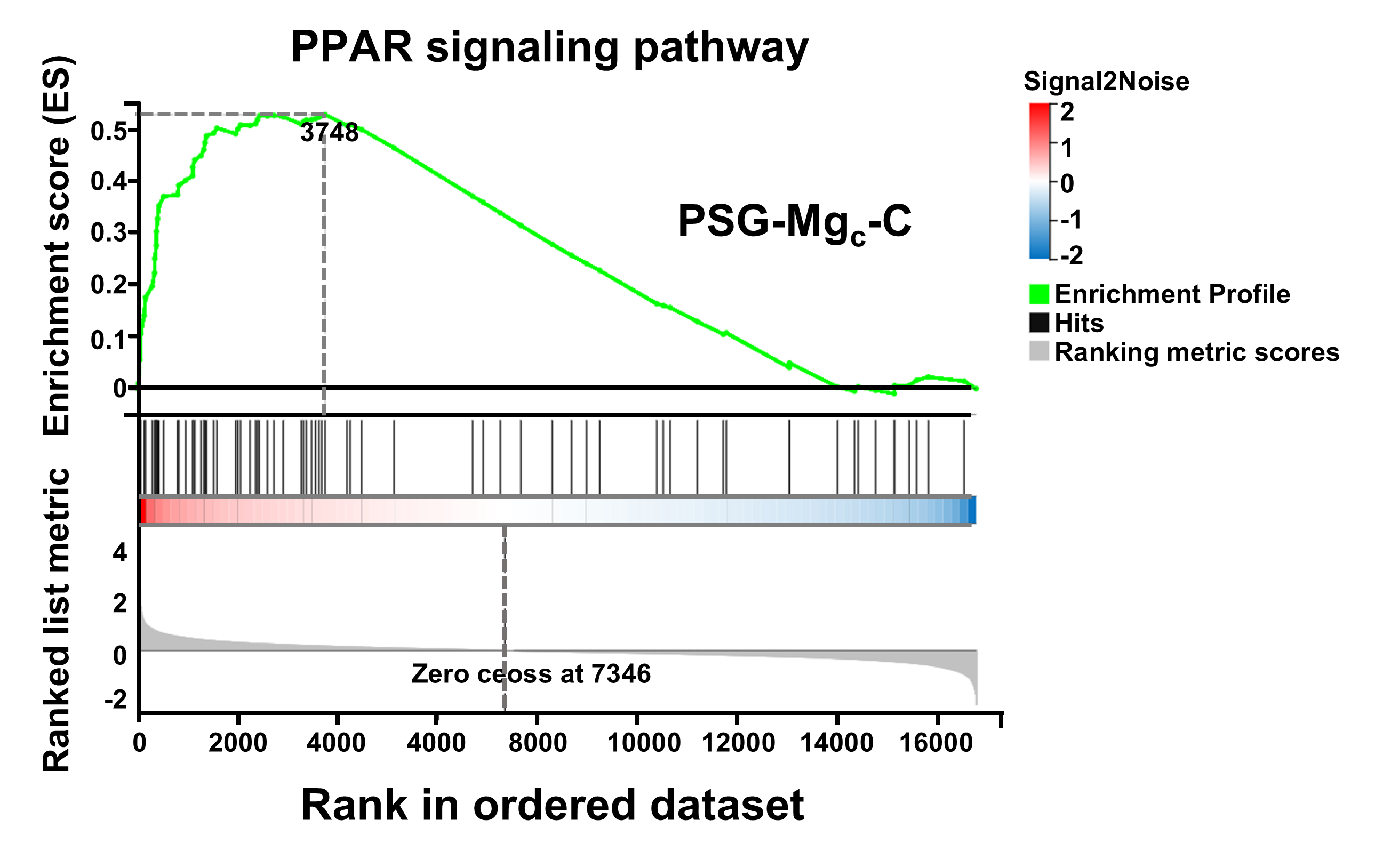


**Figure S17**. Enrichment analysis of GSEA genes in PSG-Mg_c_-C group regarding the PPAR signaling pathway.


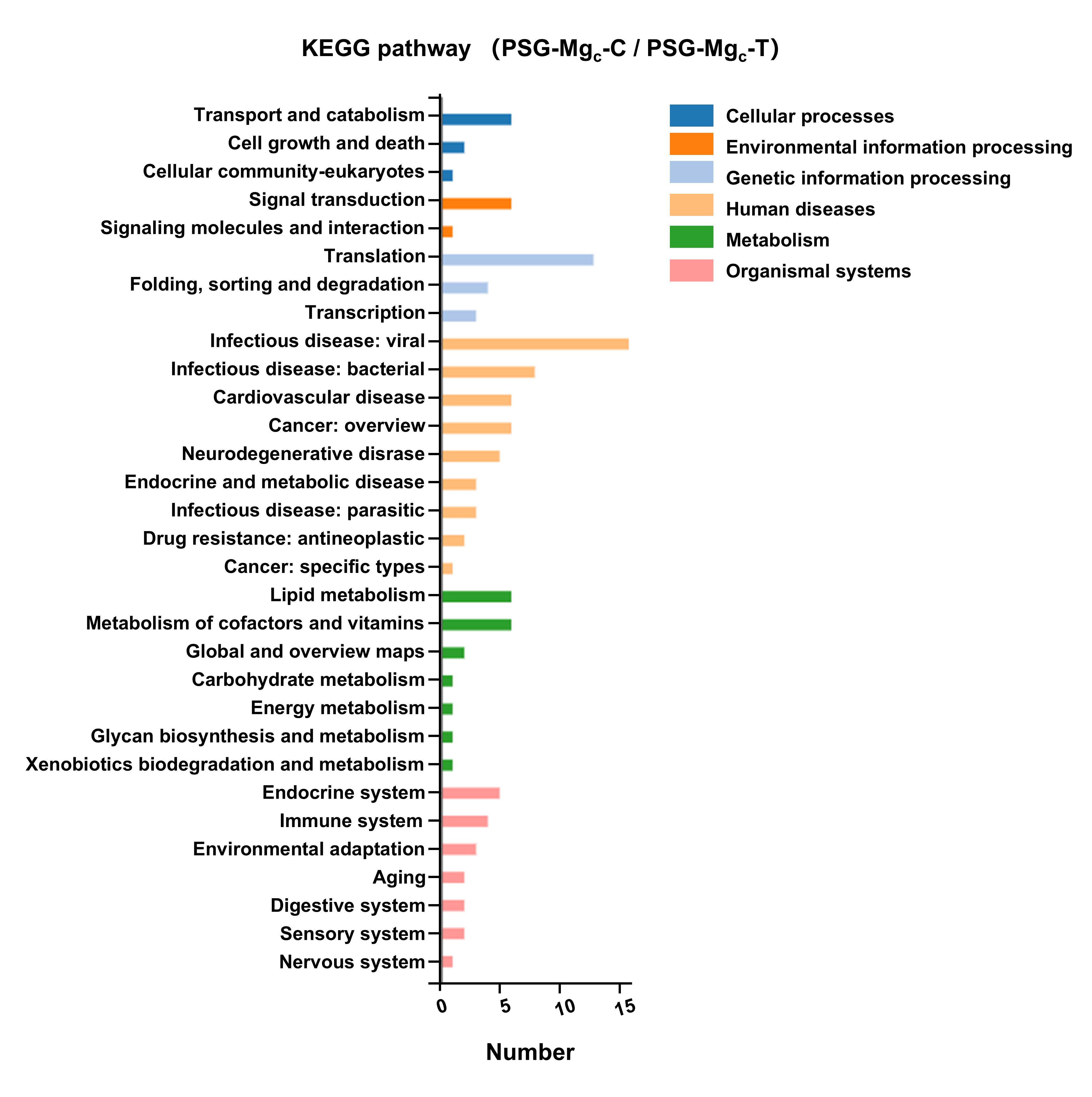


**Figure S18.** Comparative KEGG pathway analysis of neo-cartilage tissues from femoral condylar (PSG-Mg_c_-C) and trochlea defects (PSG-Mg_c_-T) implanted with the hydrogels, focusing on Cellular processes, Environmental information processing, Genetic information processing, Human disease, Metabolism, and Organismal systems.


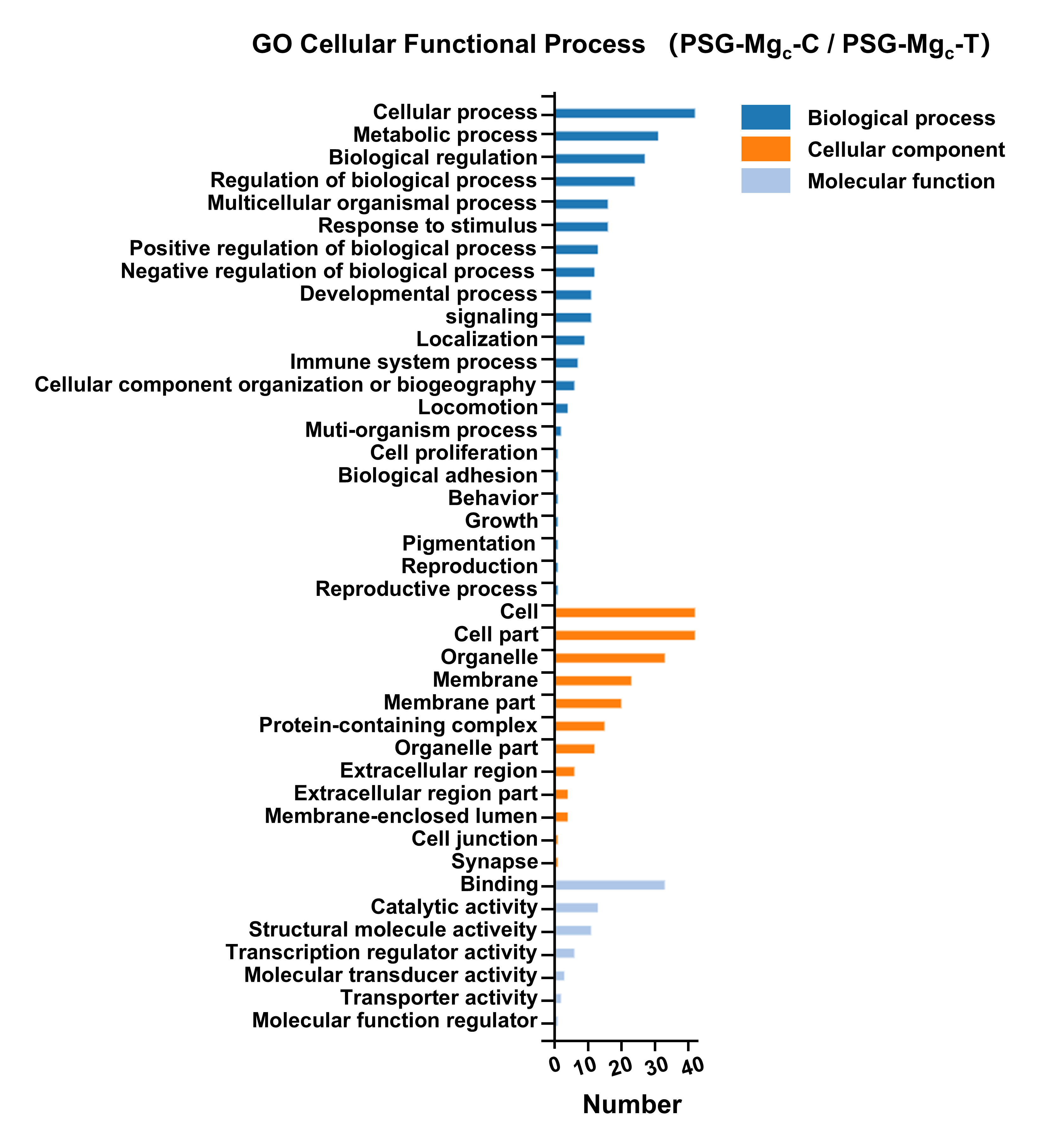


**Figure S19.** Comparative GO cellular functional process of neo-cartilage tissues from femoral condylar (PSG-Mg_c_-C) and trochlea defects (PSG-Mg_c_-T) implanted with the hydrogels, focusing on Biological process, Cellular component, and Molecular function.

1. **Supplementary Tables**

**Table S1** Composition of composite hydrogels

| **Component** | | **PVA+SA (PS)** | **PVA+Acy-Gel (PG)** | **PVA+SA+Acy-Gel (PSG)** |
| --- | --- | --- | --- | --- |
| PVA | 10% | 10% | 10% |  |
| SA | 4% | N/A | 2% |  |
| Acy-Gel | N/A | 4% | 2% |  |
| Tween 80 | 5% | 5% | 5% |  |

**Table S2** The primer sequences for qRT-PCR analysis of related genes.

| Genes | 5’-3’ | Primers |  |
| --- | --- | --- | --- |
| SOX9 | Forward primers | GGTGCTCAAGGGCTACGACT | |
|  | Reverse primers | GGGTGGTCTTTCTTGTGCTG | |
| COL-II | Forward primers | TGAAGACACCAAGGACTGCC | |
|  | Reverse primers | GCAGTGGCGAGGTCAGTAG | |
| MAGT1 | Forward primers | GGCGATATTCCAGTGCATTT | |
|  | Reverse primers | GTTGGAGCCGAATTCATGTT | |
| CAMK2D | Forward primers | CAGTACATGGATGGCAGTGG | |
|  | Reverse primers | AGGTGGCTTGATGGGTACTG | |
| CALM1 | Forward primers | AAGAGGAGATCCGTGAAGCA | |
|  | Reverse primers | AGCTTTTCCCCCAAGTTTGT | |
| PIEZO1 | Forward primers | CTGCTGCTGCACATCATTTT | |
|  | Reverse primers | ACATGCCGATGTCAGGTACA | |
| GAPDH | Forward primers | TCACCATCTTCCAGGAGCGA | |
|  | Reverse primers | CACAATGCCGAAGTGGTCGT | |

**Table S3.** Description of experimental grouping at 8- week.

(“Left” is abbreviated as “L” and “Right” is abbreviated as “R”

| **Trochlea defect** | | | **Condyle defect** | | **Control** | | | **Sham** | | |
| --- | --- | --- | --- | --- | --- | --- | --- | --- | --- | --- |
| **4 rabbits** | | | **2 rabbits** | | **4 rabbits** | | | **4 rabbits** | | |
| **number** | **PSG_c_-T** | **PSG-Mg_c_-T** | **number** | **PSG-Mg_c_-C** | **number** | **Trochlea defect** | **Condyle defect** | **number** | **Trochlea** | **Condyle** |
| **1** | L | R | **5** | L&R | **7** | L&R |  | **11** | L&R |  |
| **2** | L | R | **6** | L&R | **8** | L&R |  | **12** | L&R |  |
| **3** | L | R |  |  | **9** |  | L&R | **13** |  | L&R |
| **4** | L | R |  |  | **10** |  | L&R | **14** |  | L&R |

**Table S4.** Experimental grouping of animal models for RNA-seq of regenerated cartilage tissue.

(“Left” is abbreviated as “L” and “Right” is abbreviated as “R”

| **Trochlea defect** | | | **Condyle defect** | |
| --- | --- | --- | --- | --- |
| **4 rabbits** | | | **2 rabbits** | |
| **number** | **PSG_c_-T** | **PSG-Mg_c_-T** | **number** | **PSG-Mg_c_-C** |
| **1** | L | R | **5** | L&R |
| **2** | L | R | **6** | L&R |
| **3** | L | R |  |  |
| **4** | L | R |  |  |

**Table S5.** Mankin Score

| **Category** | **Item** | **Points** |
| --- | --- | --- |
| Structure of cartilage | Normal | 0 |
|  | Surface irregularities | 1 |
|  | Pannus and surface irregularities | 2 |
|  | Clefts to transitional zone | 3 |
|  | Clefts to radial zone | 4 |
|  | Clefts to calcified zone | 5 |
|  | Complete disorganization | 6 |
| Chondrocytes | Normal | 0 |
|  | Increase or slight decrease | 1 |
|  | Moderate decrease | 2 |
|  | Severe decrease | 3 |
|  | No cells | 4 |
| Cartilage matrix staining (Saf-O) | Normal | 0 |
|  | Slight decrease | 1 |
|  | Moderate decrease | 2 |
|  | Severe decrease | 3 |
|  | No staining | 4 |
| Tidal line integrity | Normal | 0 |
|  | Disappearance or invasion by vessels | 1 |
| **Total points** | | **15** |

**Table S6.** Wakitani Score

| **Category** | **Item** | **Points** |
| --- | --- | --- |
| Cell morphology | Hyaline cartilage | 0 |
|  | Mostly hyaline cartilage | 1 |
|  | Mostly fibrocartilage | 2 |
|  | Mostly non-cartilage | 3 |
|  | Non-cartilage only | 4 |
| Matrix staining | Normal | 0 |
|  | Slightly reduced | 1 |
|  | Markedly reduced | 2 |
|  | No metachromatic stain | 3 |
| Surface regularity (total smooth area compared with entire area of cartilage defect) | Smooth (> 3/4) | 0 |
|  | Moderate (> 1/2 - 3/4) | 1 |
|  | Irregular (1/4 - 1/2) | 2 |
|  | Severely irregular (< 1/4) | 3 |
| Thickness of cartilage | > 2/3 | 0 |
|  | 1/3 - 2/3 | 1 |
|  | < 1/3 | 2 |
| Structural integrity | Both edges integrated | 0 |
|  | One end integrated | 1 |
|  | Neither edge integrated | 2 |
| **Total points** | | **14** |

1. **Supplementary Reference**

[1] R. Shannon, "Revised effective ionic radii and systematic studies of interatomic distances in halides and chalcogenides," *Acta Crystallographica Section A: Foundations and Advances* A32 (1976): 751-767.

[2] P. Tordi, F. Ridi, P. Samorì, and M. Bonini, "Cation-alginate complexes and their hydrogels: A powerful toolkit for the development of next-generation sustainable functional materials," *Advanced Functional Materials* 35 (2025): 2416390.

[3] F. Abasalizadeh, S. V. Moghaddam, E. Alizadeh, et al., "Alginate-based hydrogels as drug delivery vehicles in cancer treatment and their applications in wound dressing and 3D bioprinting," *Journal of Biological Engineering* 14 (2020): 8.

[4] F. Topuz, A. Henke, W. Richtering, and J. Groll, "Magnesium ions and alginate do form hydrogels: a rheological study," *Soft Matter* 8 (2012): 4877.

[5] X. Wang, T. Zhang, W. Dai, et al., "Co-delivering GAG/Mg-GA MOF-through core-shell nanofibers for enhanced osteoarthritis cartilage regeneration," *Chemical Engineering Journal* 513 (2025): 163019.

[6] C. Gao, W. Dai, X. Wang, et al., "Magnesium gradient-based hierarchical scaffold for dual-lineage regeneration of osteochondral defect," *Advanced Functional Materials* 33 (2023): 2304829.

[7] C. Gao, W. Dai, D. Liu, et al., "Adaptive hydrogel loaded with pre-coordinated stem cells for enhanced osteoarthritis therapy," *Bioactive Materials* 51 (2025): 613-633.

[8] W. Wang, and K. W. K. Yeung, "Bone grafts and biomaterials substitutes for bone defect repair: A review," *Bioactive Materials* 2 (2017): 224-247.
